# Systematically Defined Informative Priors in Bayesian Estimation: An Empirical Application on the Transmission of Internalizing Symptoms Through Mother-Adolescent Interaction Behavior

**DOI:** 10.3389/fpsyg.2021.620802

**Published:** 2021-03-24

**Authors:** Susanne Schulz, Mariëlle Zondervan-Zwijnenburg, Stefanie A. Nelemans, Duco Veen, Albertine J. Oldehinkel, Susan Branje, Wim Meeus

**Affiliations:** ^1^Youth and Family, Utrecht University, Utrecht, Netherlands; ^2^Methodology and Statistics, Utrecht University, Utrecht, Netherlands; ^3^Julius Global Health, Julius Center for Health Sciences and Primary Care, University Medical Center Utrecht, Utrecht, Netherlands; ^4^Optentia Research Program, North-West University, Potchefstroom, South Africa; ^5^Interdisciplinary Center Psychopathology and Emotion Regulation, University of Groningen, University Medical Center Groningen, Groningen, Netherlands

**Keywords:** intergenerational transmission, internalizing psychopathology, mother-adolescent interaction, informative priors, linear pool, logarithmic pool, Bayesian estimation, longitudinal mediation analysis

## Abstract

**Background:**

Bayesian estimation with informative priors permits updating previous findings with new data, thus generating cumulative knowledge. To reduce subjectivity in the process, the present study emphasizes how to systematically weigh and specify informative priors and highlights the use of different aggregation methods using an empirical example that examined whether observed mother-adolescent positive and negative interaction behavior mediate the associations between maternal and adolescent internalizing symptoms across early to mid-adolescence in a 3-year longitudinal multi-method design.

**Methods:**

The sample consisted of 102 mother-adolescent dyads (39.2% girls, *M*_*age*_ T1 = 13.0). Mothers and adolescents reported on their internalizing symptoms and their interaction behaviors were observed during a conflict task. We systematically searched for previous studies and used an expert-informed weighting system to account for their relevance. Subsequently, we aggregated the (power) priors using three methods: linear pooling, logarithmic pooling, and fitting a normal distribution to the linear pool by means of maximum likelihood estimation. We compared the impact of the three differently specified informative priors and default priors on the prior predictive distribution, shrinkage, and the posterior estimates.

**Results:**

The prior predictive distributions for the three informative priors were quite similar and centered around the observed data mean. The shrinkage results showed that the logarithmic pooled priors were least affected by the data. Most posterior estimates were similar across the different priors. Some previous studies contained extremely specific information, resulting in bimodal posterior distributions for the analyses with linear pooled prior distributions. The posteriors following the fitted normal priors and default priors were very similar. Overall, we found that maternal, but not adolescent, internalizing symptoms predicted subsequent mother-adolescent interaction behavior, whereas negative interaction behavior seemed to predict subsequent internalizing symptoms. Evidence regarding mediation effects remained limited.

**Conclusion:**

A systematic search for previous information and an expert-built weighting system contribute to a clear specification of power priors. How information from multiple previous studies should be included in the prior depends on theoretical considerations (e.g., the prior is an updated Bayesian distribution), and may also be affected by pragmatic considerations regarding the impact of the previous results at hand (e.g., extremely specific previous results).

## Introduction

New studies and analyses in social sciences are theoretically and empirically grounded in previous knowledge that has often accumulated in decades of research. While there is overall agreement that this process is essential to generate strong hypotheses, findings from previous studies are rarely integrated into new analyses. Accounting for such previous findings in subsequent analyses by means of informative priors in Bayesian estimation allows researchers to draw more precise conclusions and obtain insight into the relation between previous knowledge and the current data.

Bayesian estimation with informative priors increases the precision of the posterior distributions by updating previous information with new data and thus gradually accumulating knowledge. While the frequentist approach regards parameters of interests as unknown, but assumes that there is only one true parameter value in the population, the Bayesian approach regards parameters of interest as uncertain and describes them with a probability distribution (van de Schoot et al., 2014). By combining previous information with new data from the analyses, Bayesian estimation allows researchers to make assumptions about model parameters, such as curtailing or excluding certain parameter values (Zondervan-Zwijnenburg et al., 2017). To date, most empirical studies rely on diffuse or naive prior distributions, such as default software settings, that do not account for the available previous knowledge (e.g., van de Schoot et al., 2017). Simulation studies and mathematical demonstrations indicated that using informative priors that are derived from previous studies, meta-analyses, or experts, outperformed frequentist approaches and approaches using diffuse priors in terms of decreased relative bias, improved estimation accuracy (e.g., decreased RMSE values), and increased power when samples were too small for complex analyses (Smid et al., 2019; Zitzmann et al., 2020). However, if informative priors are not chosen carefully or are weakly defined, Bayesian estimation methods may perform poorly and result in biased estimates (Depaoli, 2013; Holtmann et al., 2016). Therefore, a systematic and transparent approach is essential when specifying informative priors (Zondervan-Zwijnenburg et al., 2017; van de Schoot et al., 2021). The present study highlights the use of different approaches to systematically define informative priors and the integration of new data to answer novel research questions.

### Weighting Previous Studies

If previous designs are not consistent with the new study, for example due to different populations or different assessments, this can raise potential bias and inflated type I errors (Hobbs et al., 2011; Viele et al., 2014). Previous findings should therefore strongly inform the posterior distributions when they are based on designs that are comparable to the present study, and weakly when they are not. To ensure that previous findings do not outweigh the current data and dominate the posterior distributions, power priors that downweigh previous data by determining the amount of relevant information have been recommended (Ibrahim and Chen, 2000). Specifically, a power prior takes the likelihood of the information from the previous study to the power δ, where δ is a value between 0 (ignore the previous data completely) and 1 (treat the data as equal to the current data and fully include the evidence). For normal distributions, when delta δ≠ 0, raising the likelihood to the power δ is equal to dividing the variance from the previous study by δ and using it as the prior variance $\sigma_{0}^{2}$. Traditionally, power priors include the use of unknown weights, which have been criticized to over-attenuate the influence of previous data (Neelon and O’Malley, 2010) as they do not capture the extent to which previous findings are applicable to the present design and data.

Previous studies can be more or less similar to a specific study’s design and thus provide stronger or weaker input for priors than other studies. Meta-analyses, for example, quantify existing information, and thus provide accumulated, more robust evidence than single studies. However, they also include a wide range of different methodological designs, such as different participant age ranges or assessment methods, and thus cannot provide strong input for specific parameter estimates if individual participant data is not available. Empirical studies that closely reflect the research questions and design of the new study that is to be conducted provide the strongest input for informative priors, but are more susceptible to potential estimation errors, biases, or chance findings than meta-analyses. How much weight a particular study receives, should therefore depend on a range of aspects that correspond to the study’s design at hand. Longitudinal studies, for example, involve different considerations than cross-sectional studies, such as temporal ordering and the lengths of intervals between time points. If the study at hand employs a longitudinal design, findings from studies with repeated measurements would receive more weight than studies that solely include measurements at the same time point. Only a previous study with data that can be considered exchangeable with the new data should receive a weight of 1. To determine how much an individual study deviates from the new data, we therefore propose to determine each study’s individual weight for the construction of power priors. Studies with lower relevance obtain lower scores for δ, which means that their variance will be inflated. The larger the variance (i.e., uncertainty), the smaller the impact of a previous study on the specified prior distribution, and therefore also on the posterior distribution.

A carefully constructed and justified weighting scheme that is tailored to the specific research question is essential when specifying informative priors. To avoid arbitrary and subjective decisions, expert knowledge can inform this process (Bolsinova et al., 2017; van de Schoot et al., 2018; Veen et al., 2020). Expert knowledge as input for prior distributions has been previously used to estimate the size of parameters for which no data was available (e.g., Hald et al., 2016) or to complement existing data (e.g., van de Schoot et al., 2018). Our proposed method includes quantifying and weighing previous information, systematically collecting and justifying all decisions, visualizing informative priors, and conducting sensitivity analyses to compare the impact of different priors on the posterior estimates (Zondervan-Zwijnenburg et al., 2017). This can be beneficial beyond a pure meta-analytical approach that solely quantifies previous information. As such, Bayesian estimation with informative priors allows researchers to update previous information by combining it with new data. This cumulative process gradually decreases the uncertainty of parameter estimates (König and van de Schoot, 2018). In the current study, we used expert knowledge to define inclusion criteria and create an appropriate weighting scheme for all included previous studies.

### Aggregating Previous Studies

If multiple studies contain information on one parameter, the previous information needs to be aggregated into one distribution. Three aggregation methods are: (1) linear pooling, (2) logarithmic pooling, and (3) a normal distribution fitted to the linear pool.

#### Linear Pooling

The linear pool of distributions sums the densities provided by the different studies, resulting in a mixture prior (Genest and Zidek, 1986). The linear pool directly represents the previous studies by combining them without any modifications to the initial information. One way to obtain the linear pool is to run multiple Bayesian analyses: one for each prior specification. Subsequently, the posterior samples can be aggregated (see Zondervan-Zwijnenburg et al., 2017). This method can be applied in any software package that allows for Bayesian estimation with customizable prior specifications. However, as parameter estimates within a model are not independent, this method becomes impractical in a model in which multiple parameters have multiple sources of previous information. In more advanced Bayesian software such as Stan (Carpenter et al., 2017), the linear pool of previous studies can be programmed at once. A difficulty that remains is that a linear pool becomes multimodal when the different prior likelihoods diverge. Multimodality is complex for estimation and interpretation. It may cause non-convergence, and it can be odd to consider, for example, 0.2 and 0.5 equally plausible values, but 0.35 a value with low probability. There is the possibility that this scenario occurs when local maxima have previously been found.

#### Logarithmic Pooling

Whereas the linear pool sums distributions, the logarithmic (a.k.a. geometric) pool multiplies them. In practice this means that extreme modes originating from only one study can be compensated by their multiplication with other studies that allocate less probability to this area. In this manner, the logarithmic pool emphasizes the common range of parameter values. Logarithmic pools are typically unimodal and less dispersed than linear pools (Genest and Zidek, 1986). The logarithmic pool can also be considered a Bayesian updating procedure, in which the first^1^ study is the initial prior. A potential disadvantage of the logarithmic pool, however, is that if one previous study places near-zero probability to a range of values, the multiplication by near-zero probability will predominate in the pooled distribution. de Carvalho et al. (2020) define pooled distribution and their parameters for sets of common distributions. When the pooled distribution is a common distribution as well, the prior can be easily specified in software packages that allow for Bayesian estimation with custom prior distributions.

#### Normal Distribution Fitted to the Linear Pool

Another alternative to including a potentially bimodal linear pool, is to obtain the normal distribution best fitting to this pooled distribution. In this method, the previous studies are considered to be samples from an underlying normal distribution. By fitting a normal distribution to the results of the previous studies, we aim to retrieve the underlying normal distribution of the parameter. When the underlying previous studies have different means, the fitted normal distribution will have a variance larger than that of the underlying studies. Once the hyperparameters of the fitted normal distribution are obtained, the normal prior distribution can be specified in any software package that allows for Bayesian estimation with custom prior distributions.

Conducting sensitivity analyses with different priors, including diffuse default priors, allows us to compare findings and highlight the robustness of our model results if priors are modified (van Erp et al., 2018). The current study will compare the results of these three pooling methods and diffuse default priors on the posterior distributions in an empirical example that examined whether mother-adolescent interaction behavior meditates the associations between maternal and adolescent internalizing behavior.

### Empirical Application: Mother-Adolescent Interaction Behavior as Mediator in the Transmission of Internalizing Symptoms

Adolescence is a crucial period for the development of internalizing problems, such as symptoms of anxiety or depression, which increase adolescents’ risk for psychopathological disorders, school dropout, and unemployment in later life (Kessler et al., 2012; Clayborne et al., 2019). Maternal internalizing symptoms are among the most salient predictors of adolescent internalizing symptoms (e.g., Goodman and Gotlib, 1999; Connell and Goodman, 2002). Genetic similarities cannot fully explain these associations (Natsuaki et al., 2014; Eley et al., 2015) and specific patterns of how mothers and adolescents interact may be another mechanism through which maternal internalizing symptoms are associated with adolescent internalizing symptoms (Goodman and Gotlib, 1999). Specifically, internalizing symptoms might render mothers less sensitive to their children’s needs, more emotionally unavailable, and more irritated, which can suppress mothers’ expression of positive interaction behavior and increase their expression of negative, hostile and angry interaction behavior toward the adolescent (Lovejoy et al., 2000). Such diminished positive and heightened negative interaction behavior may in turn undermine the adolescents’ self-esteem and emotion-regulation, make them feel helpless, and prompt negative self-evaluations, which render them more sensitive to internalizing symptoms (Gottman et al., 1997; Garber and Flynn, 2001). Hence, it is likely that maternal interaction behavior underlies the transmission of internalizing symptoms from mothers to adolescents.

Transactional theories (e.g., Sameroff, 2009) suggest that adolescents are not only influenced by their parents, but also influence their parents. Hence, associations between maternal and adolescent internalizing symptoms are likely to be bidirectional (Hughes and Gullone, 2010; Wilkinson et al., 2013). Adolescent internalizing symptoms can disrupt interactional processes in the family (Sheeber et al., 2001; Berg-Nielsen et al., 2002) and thus, similarly, predict changes in mother-adolescent interaction behavior (e.g., Nelemans et al., 2014), which in turn prompt maternal internalizing symptoms. It is thus important to include bidirectional associations between maternal and adolescent internalizing symptoms when investigating the mediating role of mother-adolescent interaction behavior. Similarly, as social interactions include two partners who continuously regulate and react to each other’s behaviors (Fogel, 1993), it is essential to examine not only maternal interaction behavior toward adolescents, but also adolescents’ interaction behavior toward mothers. However, most studies to date are based on the assumption that associations between maternal and adolescent internalizing symptoms are unidirectional from mothers to adolescents and only driven by maternal interaction behavior toward adolescents. If potential effects from adolescents to mothers are ignored, alleged mediation effects may be spurious. Fully understanding the mediating role of mother-adolescent positive and negative interaction behavior in the transmission of internalizing symptoms thus requires a model that reflects reciprocal associations between mothers and adolescents. In this study, we will investigate whether mother-adolescent interaction behavior underlies the intergenerational transmission of internalizing symptoms, including associations from both mothers to adolescents and from adolescents to mothers.

Several studies have been conducted to support each pathway in the theoretically proposed mediation model (see Supplementary Material for a systematic and critical review of previous literature). Findings from meta-analyses on mother-child interactions indeed indicated associations of maternal interaction behavior with maternal internalizing symptoms (Lovejoy et al., 2000; McCabe, 2014) and child internalizing symptoms (McLeod et al., 2007a,b; Yap et al., 2014; Pinquart, 2017). Observational, longitudinal assessments in adolescence best reflect our study’s design and thus provide strong specific information. The few studies that meet these criteria, however, remain inconsistent regarding whether maternal internalizing predict both subsequent positive (Simons et al., 1993; Feng et al., 2007) and negative interaction behavior (Feng et al., 2007) as well as whether interaction behavior predicts subsequent adolescent internalizing symptoms (Hofer et al., 2013; Milan and Carlone, 2018) or not (Feinberg et al., 2007; Schwartz et al., 2012). Studies on reversed associations from adolescents to mothers remain scarce and the one available study found that adolescent interaction behavior did not predict maternal internalizing symptoms (Milan and Carlone, 2018).

### The Present Study

**FIGURE 1:**
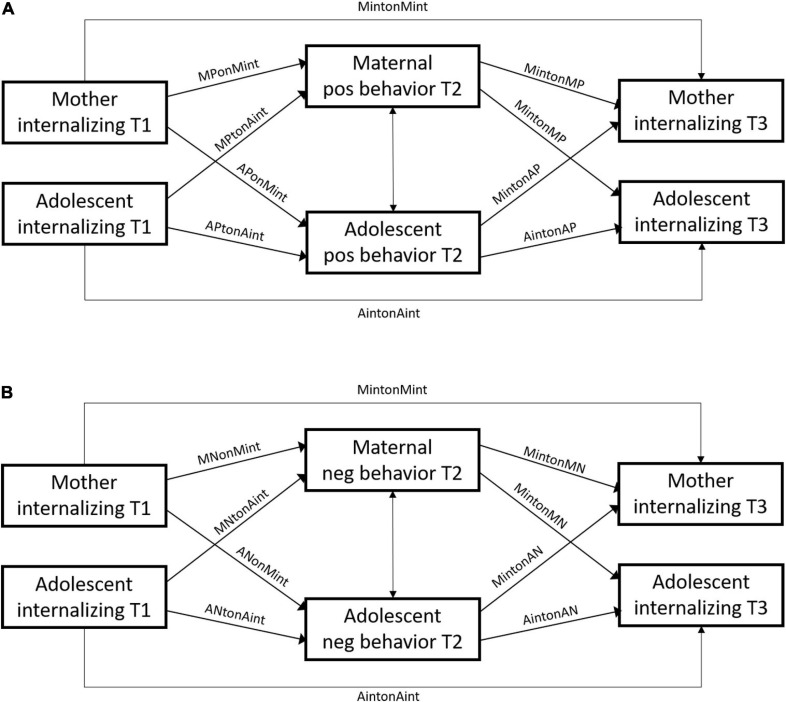
Conceptual SEM models examining the mediating effects of positive (model **A**) and negative interaction behavior (model **B**) in the associations between maternal and adolescent internalizing symptoms. M, maternal; A, adolescent; pos, positive; neg, negative.

This study applied a systematic approach to defining informative priors in Bayesian estimation to highlight the role of Bayesian estimation in integrating and cumulating empirical knowledge. We compared the effects of three different kinds of informative priors on the posterior distribution using an empirical illustration: Specifically, we examined whether observed mother-adolescent positive and negative interaction behavior mediate associations between maternal and adolescent internalizing symptoms, using a multi-method longitudinal design (see Figure 1). To increase the precision of our results, we systematically searched and weighed findings from previous studies, using an expert-designed weighting and scoring system, and synthesized the information into linear pool, logarithmic pool, and fitted normal prior distributions. Furthermore, we conducted sensitivity analyses to compare the impact of informative and diffuse priors on the mediating effects of mother-adolescent interaction behavior in the transmission of internalizing symptoms. This allowed us to identify the role of different priors and the robustness of our results.

## Materials and Methods

All relevant materials, documents, and syntax files are available at https://osf.io/c37mv.

### Participants

The sample consisted of 102 mother-adolescent dyads (39% girls, *M*_*age*_ T_1_ = 13.0, *SD*_*age*_ = 0.51) who were part of a larger sample of families participating in the ongoing Research on Adolescent Development And Relationships Young (RADAR-Y) study. All participants were assessed in annual home visits. Most adolescents (95%) and their mothers (91%) were of Dutch origin. They predominantly lived with both biological parents (86%) in medium to high socioeconomic status households (91%), based on parents’ occupation level.

Sample attrition was low across all time points (1–7%), with 94 mother-adolescent dyads who participated at the first time point remaining in the study at the third time point. Mothers and adolescents who dropped out of the study did not significantly differ from those who remained in the study on most of the study or background measures (ANOVA *p*-values ≥ 0.056). However, mothers who dropped out of the study showed more negative interaction behavior at the second time point, *F*(1,87) = 4.67, *p* = 0.033, than mothers who remained in the study.

### Procedure

The present study used three time points from early to mid-adolescence, when adolescents were on average approximately 13, 14, and 15 years of age. Families were recruited through 230 randomly selected elementary schools in the central and western regions of Netherlands. Of those initially selected (*N* = 1,544), families who did not fulfil the full family requirements (*n* = 364), could not be contacted or withdrew their participation (*n* = 569), or did not provide written consent for all family members (*n* = 114) were excluded. Of those 497 families who participated at the first time point, a subsample of 102 randomly selected mother-adolescent dyads participated in an interaction task.

During annual home assessments, adolescents and their mothers completed a series of questionnaires and subsequently participated in a conflict interaction task. The conflict task consisted of a 10-min videotaped interaction between adolescents and their mothers, during which they discussed a topic of frequent disagreement, explained their individual thoughts, and presented a solution to the conflict. Prior to the task, adolescents and their mothers agreed upon a topic, chosen out of a series of suggested subjects or an own subject. The interviewer ensured that a topic was chosen, but was otherwise absent during the topic selection and the actual conflict task. Adolescents and mothers were compensated for their participation at each time point. The study procedure was approved by the Medical Research Ethics Committee of the University Medical Center Utrecht.

### Measures

#### Adolescent Internalizing Symptoms

We assessed adolescent internalizing symptoms as a combined score of self-reported anxiety and depression symptoms. Anxiety symptoms were measured with the Screen for Child Anxiety Related Emotional Disorders (SCARED; Birmaher et al., 1997), which consists of 38 items (e.g., “I get really frightened for no reason at all”) on a 3-point scale (1 = almost never, 3 = often). Depression symptoms were measured with 2nd edition of the Reynolds Adolescent Depression Scale (RADS-2; Reynolds, 2000), which consists of 23 items (e.g., “I feel that no one cares about me”) on a 4-point scale (1 = almost never, 4 = often). As anxiety and depression symptoms correspond to the same higher-order latent factor of internalizing symptoms within a hierarchical structure of psychopathology (Achenbach, 1966; Lahey et al., 2017), total anxiety and depression scores were averaged after a multiple imputation procedure to form a total internalizing symptom score for each participant. The anxiety, depression, and total internalizing scales showed high internal consistency across all time points (α = 0.91–0.96). Higher scores indicated higher levels of adolescent internalizing symptoms.

#### Maternal Internalizing Symptoms

We assessed maternal internalizing symptoms with the anxious/depressed, withdrawn, and somatic complaints syndrome scales of the Adult Self Report (ASR; Achenbach and Rescorla, 2003). The syndrome scales consist of 18 items (e.g., “I feel lonely”), 9 items (e.g., “I keep from getting involved with others”), and 12 items (e.g., “I feel tired without good reason), respectively, that are measured on a 3-point scale (0 = not true, 2 = very true or often true). The total internalizing scale showed high internal consistency across all time points (α = 0.90–0.91). Higher scores indicated higher levels of maternal internalizing symptoms.

#### Maternal and Adolescent Interaction Behavior

Rating scales were adapted from the *Family Interaction Task* coding system (Weinfield et al., 1999, 2002). We observed maternal and adolescent positive interaction behavior toward the other by coding verbal and nonverbal expressions/displays of maternal emotional involvement during the conflict task. Verbal expressions include showing interest, listening, responding, and understanding. Nonverbal expressions included smiling, interested attitude, nodding, maintained eye contact. We observed maternal and adolescent negative interaction behavior toward the interaction partner by coding how hostile and angry the mother or adolescent behaved during the conflict task. Maternal negative behaviors included blaming, rejecting, mocking, and exerting negative facial expressions or physical reactions. Adolescent negative behaviors included sighing and groaning, pouting, refusing to cooperate, criticizing, and exerting negative facial expressions or physical reactions.

Three independent raters coded maternal and adolescent interaction behavior toward the other on a 5-point scale (1 = low score on the relevant interaction behavior, 5 = high score on the relevant interaction behavior). All raters underwent extensive training before coding a random selection of the sample. Higher scores of positive interaction behavior indicated more common, appropriate, and consistent use of these verbal and nonverbal expressions, while higher scores of negative interaction behavior indicated higher levels of negative, hostile behaviors. Interrater agreements using intraclass correlations (ICC) based on 15% of the sample showed acceptable agreement for maternal interaction behavior (ICCs = 0.80–0.89) and adolescent interaction behavior (ICCs = 0.86–0.87).

### Prior Distributions From Previous Knowledge

**FIGURE 2:**
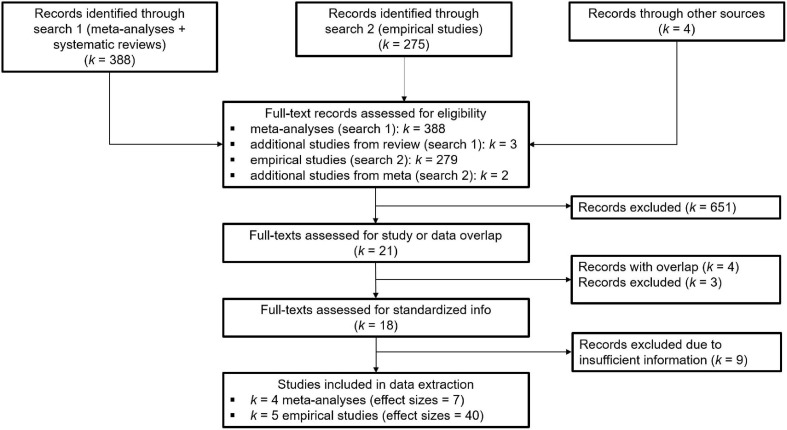
Flow chart for study inclusion from search 1 (meta-analyses and systematic reviews) and search 2 (empirical studies) based on the PRISMA guidelines.

For the regression paths in our models, we implemented two search strategies (see Figure 2 for a flowchart on study inclusion): a search for meta-analyses and reviews, and a search for empirical studies.

#### Meta-Analyses and Systematic Reviews

We conducted a literature search in Web of Science for all meta-analyses and systematic reviews published until December 2019, based on a combination of key words that reflected the target sample (child, adolescent) and their parents (parent^∗^, maternal, and mother), internalizing symptoms (anxi^∗^, depress^∗^, and internalizing), as well as positive and negative behaviors (positive, negative, affect, warmth, hostil^∗^, and rejection) during the interaction (interaction^∗^, relation^∗^, and parenting). Meta-analyses were selected if they (a) included studies on adolescence, and (b) assessed positive and/or negative interaction behavior, as defined for our sample, from mother or parent toward adolescent and/or from adolescent toward mother or parent. This search strategy identified 388 studies, of which 7 meta-analyses and 1 systematic review were included in this study. Some meta-analyses showed substantial overlap in studies. In these cases, we only included the meta-analysis that scored highest on the scoring scheme (i.e., most comparable to our design) to avoid biasing the results. This led to a final inclusion of 4 meta-analyses, of which 2 focused on the associations between maternal internalizing symptoms and mother-adolescent interaction behavior and 2 focused on the associations between mother-adolescent interaction behavior and adolescent internalizing symptoms. The systematic review that was included identified 3 additional empirical studies that were not included in the meta-analyses and mainly focused on associations that were not investigated in any meta-analysis (e.g., associations between adolescent internalizing symptoms and adolescent interaction behavior). One of these empirical studies did not provide standardized information and was thus excluded.

#### Empirical Studies

Our second search strategy to identify relevant studies was twofold: First, we conducted a literature search in Web of Science for all empirical studies that were not included in the meta-analyses published from January 2012^2^ until March 2020 using the same search string as for the meta-analyses, but only for adolescent samples (adolescen^∗^, youth, teen^∗^, youngst^∗^, student^∗^, emerging adult^∗^, early adult^∗^, and young adult^∗^) and observational studies (observ^∗^, code^∗^, rater, tape^∗^, task^∗^, and record^∗^). Studies were selected if they (a) included an adolescent sample, but did not include participants younger than 7 years or older than 25 years at the first measurement, (b) included longitudinal estimates for the cross-lagged parameters, and (c) assessed positive and/or negative interaction behavior from mother toward adolescent and/or from adolescent toward mother using observations. This search identified 275 studies, of which 11 were included (see Figure 2). Second, we searched all cross-sectional meta-analyses for studies that met the inclusion criteria and had estimates that were not included in the meta-analytic effect sizes. This resulted in an additional inclusion of 2 studies. Studies that failed to provide any or only partial standardized information were excluded (*k* = 8). The final inclusion yielded 47 effect sizes from 4 meta-analyses and 5 independent empirical studies (see Supplementary Table 1 for all included studies per parameter and model).

#### Power Prior Weighting Scheme

To evaluate each previous study’s contribution to our research question, we designed a scoring system that reflects each study’s weight in the specification of prior distributions. Four experts on adolescent relationships and mental health (third, fifth, sixth, and seventh author) discussed and evaluated the importance of several methodological aspects, which were further quantified to represent one score (see Table 1A). For example, a longitudinal measurement most closely reflected our study design, and therefore received a higher score than a cross-sectional measurement. The final weighting scheme included ten categories: longitudinal associations, same time lag, controlling for earlier internalizing symptoms, mother-adolescent interaction behavior assessed solely observational, age range from early to mid-adolescence (12–16), included symptoms of depression and anxiety, or anxiety only, controlling for other partner’s symptoms, controlling for other partner’s interaction behavior, community sample, and meta-analysis. The ten categories were associated with 5–20 points depending on the importance of the criterion. Each included study received the allocated number of points per category depending on whether or not they fulfilled the criteria (see Table 1B). The final score for each study determined its associated weight, δ, in the power prior.

**TABLE 1A:** Weighting scheme for informative priors.

**Category**	**Points**	**Details**
T1-T2 (longitudinal)	10	The estimates of longitudinal studies are usually smaller than those of cross-sectional studies. As our parameter are longitudinal estimates as well, longitudinal designs should receive most weight in relation other categories.
- *controlling for symptoms at T1*	20	Longitudinal studies that do not control for symptoms at T1 might have quite large estimates and cannot indicate change. As this is the most crucial aspect of longitudinal research, studies that also control for T1 symptoms should receive more weight. *Not applicable for T1 → T2 associations (deleted from final score)!*
- *Same time lag - (1 year)*	5	Studies that use the same time lag as we do are closer to our study design and thus deserve more weight.
Observation	15	The study list only includes empirical studies with observational assessments of the parent-adolescent interaction as these (multi-method) estimates are usually smaller than self-reports. However, meta-analyses often include a combination of observations and self-reports, which is difficult to disentangle. Therefore, estimates from “pure” observations should receive more weight than mixed studies (and most weight in relation to other categories as this is another main aspect of our study).
Early adolescence (12–16)	10	Some studies, and particularly the meta-analyses, used a broader age range than our study or even just adolescence (but all studies include adolescence). As our study focuses on early-mid adolescence, studies that included a similar age group should receive some more weight.
Internalizing symptoms include both anxiety and depression, or anxiety only	10	Most studies do not focus on a combination of depression and anxiety symptoms, but only include one of those symptoms (mostly depression). As we will use a combination of both, studies that include measures on internalizing symptoms or both depression and anxiety symptoms should receive more weight. *Most studies focus on mother or adolescent depression (rather than anxiety). To counterbalance that, we will also award 5 points if the study only focused on anxiety (i.e., either combined or anxiety only).*
Including covariates - *parental symptoms* - *other interaction behaviors*	5 5	If studies include other relevant covariates that might better reflect our study associations, such as parental symptoms (for T2-T3 parameters), they might receive additional weight.
Community sample (does not include clinical/diagnostic groups)	10	Many (older) studies include two subsamples, of which one is usually clinical. Therefore, the final sample includes participants who may have higher levels of internalizing symptoms than our participants. For these participants, the associations may be stronger. Thus, studies with a community sample which is closer to our sample should receive more weight.
Meta-analysis	10	Meta-analyses combine information from several studies and thus provide the most comprehensive evidence. Therefore they should receive somewhat more weight than individual studies.
**10 categories (standard 5)**	**100 (80)**	**Each study can score between 0 and 100 points (or between 0 and 80 points for T1 → T2 associations).**

**TABLE 1B:** Final scoring of all included studies.

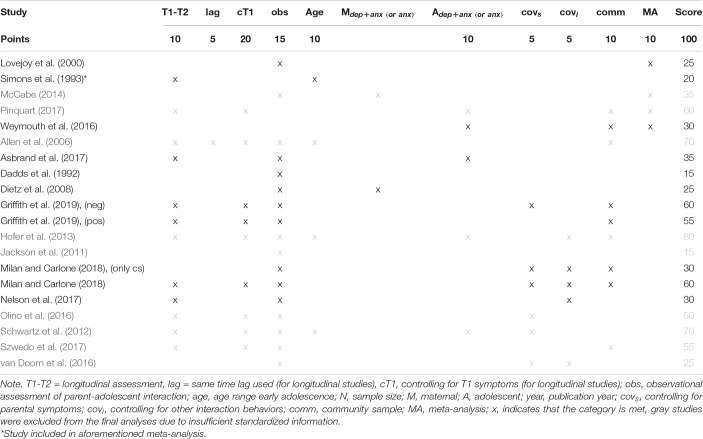

*Note.* T1-T2 = longitudinal assessment, lag = same time lag used (for longitudinal studies), cT1, controlling for T1 symptoms (for longitudinal studies); obs, observational assessment of parent-adolescent interaction; age, age range early adolescence; *N*, sample size; M, maternal; A, adolescent; year, publication year; cov_*s*_, controlling for parental symptoms; cov_*i*_, controlling for other interaction behaviors; comm, community sample; MA, meta-analysis; x, indicates that the category is met, gray studies were excluded from the final analyses due to insufficient standardized information. *Study included in aforementioned meta-analysis.

### Specification of Prior Distributions

To be able to use previous information from studies with various measures, the data of the present study was standardized, and all prior distributions concerned standardized effects. Hence, only information from previous studies that presented or allowed to compute standardized effects *and* the associated standard errors was used^3^. The hyperparameters for the normally distributed prior distributions were a mean and standard deviation.

The longitudinal associations of maternal and adolescent internalizing symptoms with mother-adolescent positive (i.e., model A) and negative interaction behavior (i.e., model B) describe the main parameters in the model (see Figure 1). We did not consider the datapoints from previous studies to be exchangeable with our current dataset, nor to be a previous sample from the same population (Spiegelhalter et al., 2004). The previous information was thus considered less relevant than the current data, and therefore, needed to be downweighed by power priors. The power prior weights δ were systematically determined through our weighting scheme (section “Power Prior Weighting Scheme”). Studies with lower relevance obtained lower scores for δ, which means that their variance was inflated. The larger variance (i.e., uncertainty) diminishes its impact on the posterior distribution.

When multiple studies contained information on one parameter, the information needed to be aggregated into one distribution. We evaluated three methods to aggregate previous information: (1) linear pooling, (2) logarithmic pooling, and (3) a normal distribution fitted to the linear pool. Additionally, we conducted sensitivity analyses with default priors from the statistical R package brms as a reference (Bürkner, 2017). The four posterior distributions were compared and evaluated based on estimation issues and interpretability to indicate the role of previous information. The defined informative priors for all longitudinal regression parameters are provided in Table 2. For all other parameters in the model, the following low-informative prior was used: *N*(0,10).

**TABLE 2:** Informative priors for the regression parameters in Model A and Model B.

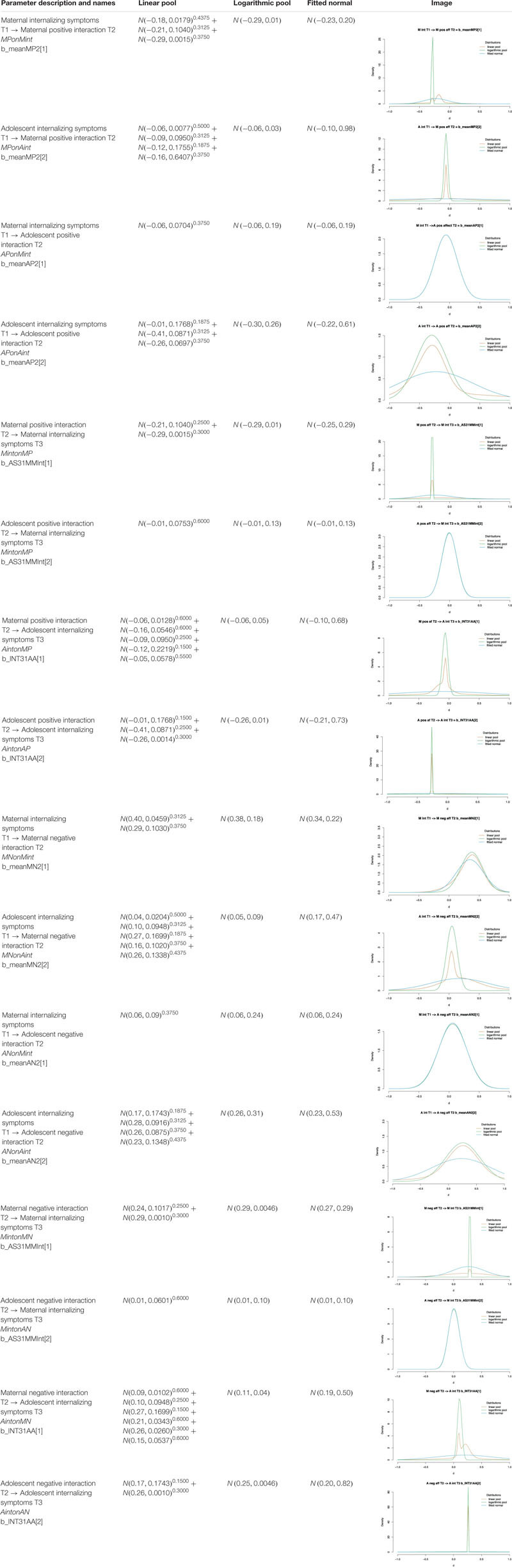

*M, maternal; A, adolescent; int, internalizing; P, positive interaction behavior; N, negative interaction behavior;on, describes the direction of regression (e.g., MPonMint indicates the association from maternal internalizing symptoms at T1 to maternal positive interaction behavior at T2). The hyperparameters of the normal distributions are a mean and a standard deviation.*

The linear and logarithmic pool both used the study’s normal prior distributions with σ/δ as input for the standard deviation. Subsequently, each of the distributions received an equal weight in the pooling procedure. The normal pool was programmed in Stan (see syntax in the Supplementary Material). The hyperparameters for the logarithmic pool of normal distributions were calculated according to de Carvalho et al. (2020). To obtain a normal distribution fitting to the linear pool, we first drew 5,000 random samples from each of the weighted normal prior distributions for one parameter. Subsequently, we fitted a normal distribution to these samples (i.e., fitted normal) by means of the fitdist function of the R-package fitdistrplus (Delignette-Muller and Dutang, 2015) using maximum likelihood estimation. The estimated mean and standard deviation associated with the best fit were used as hyperparameters for the priors in Stan.

### Statistical Analyses

Missing data was modest and ranged from 2-13% for most variables. Only at T1, 54% of the RADS-2, which is one of the two scales for internalizing problems, was missing because not all subscales of this questionnaire were administered to all participants. Based on Little’s missing completely at random (MCAR) test that detected no systematic patterns of missingness, normed χ^2^/df = 1.19, we inferred that missing data was not likely to bias our analyses. To handle the missing data, multiple imputation was conducted by means of the R-package mice (Van Buuren and Groothuis-Oudshoorn, 2011). All variables that had a correlation >0.10 with the variables to be imputed were included as predictors in the imputation model, except for the identification variable. As indicated by the imputation plots and absence of logged events, the 20 imputations were successful. The fraction of missing information (fmi) in all regression paths ranged from 0.07 to 0.38.

To evaluate the impact of the different prior distributions, we assessed convergence, conducted prior predictive checks, estimated the posterior distributions, and calculated posterior shrinkage. Convergence was assessed in randomly selected posteriors based on three imputed datasets to avoid false positives (Bürkner, 2020), using the potential scale reduction (PSR; Gelman and Rubin, 1992) and effective sample size (ESS). The PSR (or $\hat{R}$) compares the variance between and within chains. A PSR value near 1.0 indicates convergence. Originally, 1.05 was taken as an upper bound for convergence or even 1.10 with many model parameters, but more recently, smaller values like 1.01 and 1.001 have been recommended (e.g., Vehtari et al., 2019; Zitzmann and Hecht, 2019). The ESS quantifies the number of effectively independent draws from the posterior distribution, and is a measure of precision as it indicates how well an estimate is approximated. An ESS larger than 400 is recommended to get a stable estimate (e.g., Vehtari et al., 2019; Zitzmann and Hecht, 2019).

In a prior predictive check, samples are taken from the prior distribution to simulate new data based on the sampled parameter estimates. Together, the simulated datasets form the predictive distribution. The predictive distribution encompasses the data that can be expected given the multivariate prior distribution on the parameters. With a predictive distribution, the analyst can evaluate whether the (multivariate) prior relates to sensible data. Furthermore, the current observed data can be compared to the predictive distribution. In the present study, prior predictive distributions were evaluated for each of the four dependent variables and each of the four prior specifications in both models.

Posterior shrinkage (or contraction) *s* describes the degree of reduction in uncertainty from the prior to the posterior distribution of a parameter:

$$s=1-\frac{\sigma_{\text{posterior}}^{2}}{\sigma_{\text{prior}}^{2}},$$

where $\sigma_{\text{posterior}}^{2}$ is the variance of the posterior distribution and $\sigma_{\text{prior}}^{2}$ is the variance of the prior distribution. The inclusion of the likelihood of the data in the posterior tends to decrease the prior uncertainty, resulting in shrinkage. If the data is highly informative compared to the prior, the posterior shrinkage will be close to 1. If the data provides little additional information, the posterior shrinkage will be close to 0.

All Bayesian analyses were conducted in Stan by means of the rstan (Stan Development Team, 2020) and brms (Bürkner, 2017) R-packages in R 4.0 (R Core Team, 2020). We conducted our analyses with 3 chains, each running 8,000 iterations of which the first 3,000 were discarded. The software analyzed each of the 20 imputed datasets separately. Afterward, the separate posterior distributions were taken together to aggregate the results (Gelman et al., 2004, p. 520; Zhou and Reiter, 2010)^4^. We constructed two structural equation models (SEMs) to examine whether maternal and adolescent positive (see Figure 1A) and negative interaction behavior (see Figure 1B) mediated the association between maternal and adolescent internalizing symptoms across time. All models included 2-year autoregressive paths for adolescent and maternal internalizing symptoms. We further included correlations between maternal and adolescent interaction behavior. Finally, we calculated eight indirect effects to assess whether maternal and adolescent positive and negative interaction behavior mediated the associations from maternal to adolescent internalizing symptoms as well as from adolescent to maternal internalizing symptoms by multiplying the associations between internalizing symptoms and mother-adolescent interaction behavior from T1 to T2 and from T2 to T3.

## Results

### Descriptive Statistics

Table 3 displays the means, standard deviations, and correlations among all study variables. Interaction behavior correlated moderately to strongly, both within mother and adolescent interaction behavior as well as between mother and adolescent interaction behavior. Maternal and adolescent interaction behavior correlated moderately with maternal and adolescent internalizing symptoms.

**TABLE 3:** Descriptives of all study variables.

**Variable**	***M***	***SD***	**1**	**2**	**3**	**4**	**5**	**6**	**7**
1 Adolescent internalizing T_1_	–0.12	0.95							
2 Adolescent internalizing T_3_	–0.02	0.88	0.601						
3 Maternal internalizing T_1_	0.19	0.17	0.195	0.148					
4 Maternal internalizing T_3_	0.19	0.18	0.105	0.195	0.669				
5 Maternal positive interaction T_2_	3.50	0.79	–0.177	–0.293	–0.293	–0.366			
6 Maternal negative interaction T_2_	1.48	0.72	–0.81	0.082	0.184	0.185	–0.574		
7 Adolescent positive interactionT_2_	3.30	0.91	–0.185	–0.309	–0.232	–0.106	0.471	–0.260	
8 Adolescent negative interactionT_2_	1.43	0.79	0.152	0.194	0.171	0.238	–0.279	0.223	–0.735

### Convergence and Precision

PSR values were <1.01 and ESS >1,000 for all parameters in all viewed analyses for the analyses with logarithmic pooled priors, fitted normal priors, and default priors. However, the analyses with linear pooled priors also showed some insufficient results with respect to convergence and precision. In Model A, a PSR of 1.02 was observed for maternal internalizing symptoms at T1 predicting maternal positive interaction behavior at T2 (MPonMint), and a PSR of 1.04 for maternal positive interaction behavior at T2 predicting maternal internalizing symptoms at T3 (MintonMP). The ESS was <200 for maternal internalizing symptoms at T1 predicting maternal positive interaction behavior at T2 (MPonMint), maternal positive interaction behavior at T2 predicting maternal internalizing symptoms at T3 (MintonMP), and in some analyses also for adolescent positive interaction behavior at T2 predicting maternal internalizing symptoms at T3 (MintonAP). In model B, two PSR values >1.05 were observed: 1.07 for maternal negative interaction behavior at T2 predicting maternal internalizing symptoms at T3 (MintonMN), and 1.12 for adolescent negative interaction behavior at T2 predicting adolescent internalizing symptoms at T3 (AintonAN). The regression of adolescent internalizing problems on adolescent negative interaction behavior (AintonAN) was also repeatedly associated with a particularly low ESS (i.e., <50). For the purpose of this illustration, we will continue to evaluate the results as they are, without any further modifications to the estimation process.

### Prior Predictive Check

**FIGURE 3:**
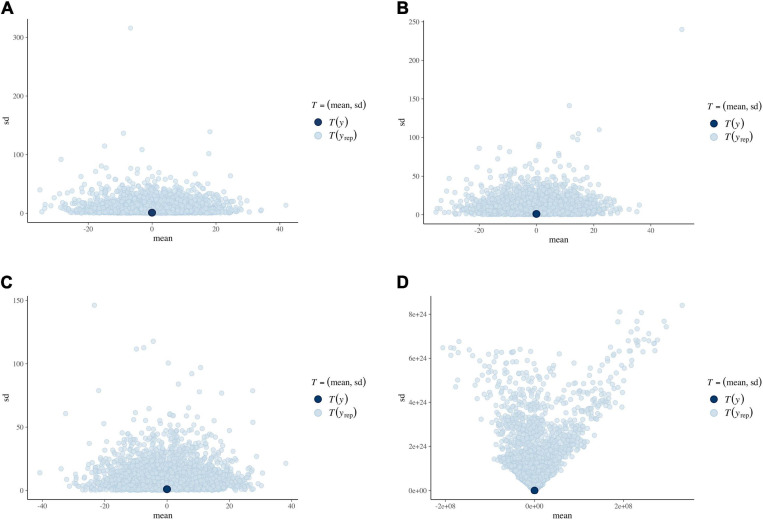
Means (*x*-axis) and standard deviations (*y*-axis) in the prior predictive distribution for the four prior specifications. The dark-blue dots represent the means in the imputed observed datasets (centered at 0). **(A)** Linear pool. **(B)** Logarithmic pool. **(C)** Fitted normal distribution. **(D)** Default.

We evaluated the predictive distributions of the four dependent model variables in both studies for each of the four methods (i.e., 32 predictive distributions). Figure 3 displays a selection of four illustrative predictive distributions.

For each of the informative prior specifications, there was a considerable spread in predicted likelihoods and their associated means and standard deviations. The predicted means mostly ranged from −40 to +40, centered around the observed data mean of 0 (all variables were centered). The predictive distribution for the default brms priors, however, almost had an infinite range including many implausible predicted likelihoods. This behavior was expected, as default priors are not supposed to direct the estimation process, but it also demonstrates that default priors do not contain meaningful information.

### Shrinkage

The posterior shrinkage for all parameters of interest and all prior specifications in both models can be found in Table 4. In all cases, the posterior shrinkage for the default brms priors was approaching 1.00, indicating that the data strongly diminished the posterior variance as compared to the prior variance. This finding was expected as default priors usually have an extremely wide variance to let the likelihood of the data predominate the posterior results. The logarithmic pool generally showed the lowest posterior shrinkage. In 9 out of 16 posterior parameter distributions, the posterior shrinkage for the logarithmic pooled prior was <0.20, and in 6 out of 16 it was even <0.05. In these cases, the logarithmic pooled prior greatly affected the posterior results. The shrinkage of the linear pooled prior and the fitted normal prior were relatively similar and varied between 0.27 and 0.90. It should be noted however, that the multimodality of the linear pooled prior and its associated posterior was not captured by our shrinkage measure that summarizes the distributions by their variances. Consequently, even though the shrinkage was larger than that of the fitted normal prior in 50% of the cases, we cannot interpret this outcome as if the likelihood had a larger impact on the posterior of the linear pooled prior than the fitted normal.

**TABLE 4:** Shrinkage in model A and B.

	**Linear pool**	**Logarithmic pool**	**Fitted normal**	**Default**
MPonMint	0.67	−0.00	0.54	> 0.99
MPonAint	0.89	0.04	0.89	> 0.99
APonMint	0.51	0.50	0.51	> 0.99
APonAint	0.83	0.60	0.81	> 0.99
MintonMP	0.54	0.00	0.66	> 0.99
MintonAP	0.27	0.39	0.32	> 0.99
AintonMP	0.89	0.10	0.84	> 0.99
AintonAP	0.82	0.01	0.84	> 0.99
MNonMint	0.57	0.49	0.57	> 0.99
MNonAint	0.82	0.20	0.77	> 0.99
ANonMint	0.58	0.58	0.58	> 0.99
ANonAint	0.81	0.67	0.80	> 0.99
MintonMN	0.58	−0.00	0.69	> 0.99
MintonAN	0.28	0.30	0.30	> 0.99
AintonMN	0.86	0.07	0.81	> 0.99
AintonAN	0.87	−0.01	0.88	> 0.99

*M, maternal; A, adolescent; int, internalizing; P, positive interaction behavior; N, negative interaction behavior;on, describes the direction of regression, indirect effects are reported in direction of the association (e.g., MintMPAint describes the indirect effect from maternal to adolescent internalizing symptoms via maternal positive interaction behavior).*

### Indirect Pathways Through Maternal and Adolescent Interaction Behavior

#### Positive Interaction Behavior

The results for the positive interaction behavior model as analyzed with the three different prior settings are provided in Table 5. Based on the analysis with linear pooled priors, we found that only for the longitudinal associations from maternal and adolescent internalizing symptoms at T1 to maternal positive interaction behavior at T2 (*M*_*maternal*_ = −0.24, 95% HPD = [−0.30,−0.13], *M*_*adolescent*_ = −0.15, 95% HPD = [−0.35,−0.04]), the 95% highest posterior density (HPD) interval did not include 0 as probable value. The completely negative 95% HPD indicates that higher levels of maternal and adolescent internalizing symptoms predicted lower levels of subsequent maternal positive interaction behavior 1 year later. Although there was limited evidence that maternal and adolescent internalizing symptoms predicted adolescent positive interaction behavior as the 95% HPD included both negative and positive values, the values were mostly negative. This indicates that there was more probability toward such a negative effect, but still some probability that the effect was positive. For all other associations, negative as well as positive values were part of the 95% HPD. Hence, we are not certain if and how positive interaction behavior at T2 predicted maternal or adolescent internalizing symptoms at T3, 1 year later. Furthermore, the 95% HPD of the autoregressive paths from maternal and adolescent internalizing symptoms at T1 to their internalizing symptoms at T3 were completely positive (*M*_*maternal*_ = 0.49, 95% HPD = [0.33,0.66], *M_*adolescent*_* = 0.44, 95% HPD = [0.27,0.61]), indicating that maternal and adolescent symptoms showed modest stability across time. All mediational paths included negative as well as positive values in their 95% HPD, indicating that there was no clear evidence on the existence and direction of the indirect effects from maternal to adolescent or adolescent to maternal internalizing symptoms through maternal or adolescent positive interaction behavior.

**TABLE 5:** Parameter estimates using different prior settings for model A.

	**Linear pool priors**			**Logarithmic pool priors**			**Normal fitted to linear pool priors**			**Default priors**		
**Parameter**	**Mean**	**95% HPD**		**Mean**	**95% HPD**		**Mean**	**95% HPD**		**Mean**	**95% HPD**	
MPonMint	–0.24	–0.30	–0.13	–0.29	–0.30	–0.28	–0.23	–0.38	–0.07	–0.23	–0.40	–0.06
MPonAint	–0.15	–0.35	–0.04	–0.07	–0.12	–0.02	–0.20	–0.38	–0.03	–0.21	–0.39	–0.03
APonMint	–0.12	–0.28	0.03	–0.13	–0.28	0.03	–0.13	–0.28	0.03	–0.15	–0.32	0.03
APonAint	–0.16	–0.33	0.01	–0.16	–0.33	0.01	–0.14	–0.33	0.05	–0.14	–0.32	0.05
MintonMP	–0.12	–0.29	0.10	–0.29	–0.30	–0.28	–0.07	–0.24	0.09	–0.04	–0.24	0.15
MintonAP	0	–0.15	0.15	0.07	–0.06	0.19	–0.02	–0.16	0.12	–0.04	–0.24	0.16
MintonMint	0.49	0.33	0.66	0.46	0.30	0.62	0.50	0.35	0.66	0.51	0.35	0.67
AintonMP	–0.06	–0.20	0.07	–0.04	–0.11	0.03	–0.08	–0.27	0.10	–0.08	–0.27	0.11
AintonAP	–0.09	–0.26	0.12	–0.26	–0.27	–0.24	–0.03	–0.23	0.16	–0.03	–0.22	0.17
AintonAint	0.44	0.27	0.61	0.42	0.24	0.59	0.45	0.27	0.61	0.45	0.28	0.61
AintMPMint	0.02	–0.01	0.08	0.02	0.01	0.04	0.01	–0.02	0.06	0.01	–0.03	0.06
AintAPMint	0	–0.03	0.03	–0.01	–0.04	0.01	0	–0.02	0.03	0.01	–0.03	0.04
MintMPAint	0.02	–0.02	0.05	0.01	–0.01	0.03	0.02	–0.02	0.07	0.02	–0.02	0.08
MintAPAint	0.01	–0.02	0.05	0.03	–0.01	0.07	0	–0.02	0.04	0	–0.03	0.04

*M, maternal; A, adolescent; int, internalizing; P, positive interaction behavior; N, negative interaction behavior; on, describes the direction of regression, indirect effects are reported in direction of the association (e.g., MintMPAint describes the indirect effect from maternal to adolescent internalizing symptoms via maternal positive interaction behavior).*

The analyses based on logarithmic pooled priors showed generally similar results. As for the analyses with the linear pooled priors, both higher levels of maternal and adolescent internalizing symptoms at T1 predicted lower levels of maternal positive interaction behavior at T2 (*M*_*maternal*_ = −0.29, 95% HPD = [−0.30,−0.28], *M*_*adolescent*_ = −0.07, 95% HPD = [−0.12,−0.02]). In contrast to the linear pooled priors, lower levels of maternal and adolescent positive interaction behavior at T2 predicted higher levels of their own, but not the other’s internalizing symptoms at T3 (*M*_*maternal*_ = −0.29, 95% HPD = [−0.30,−0.28]; *M*_*adolescent*_ = −0.26, 95% HPD = [−0.27,−0.24]). For all other direct associations, the 95% HPD included both positive and negative values. Similar to the linear pooled priors, maternal and adolescent internalizing symptoms at T1 predicted their own respective symptoms at T2. Furthermore, maternal positive interaction behavior mediated the association between adolescent and maternal internalizing symptoms, as indicated by the 95% HPD of the indirect effect that was completely positive (*M*_*indirect*_ = 0.02, 95% HPD = [0.01,0.04]). This suggests that higher levels of adolescent internalizing symptoms predicted higher levels of maternal internalizing symptoms 2 years later through decreased positive maternal interaction behavior. No other indirect effects were found.

Based on the analysis with normal distributions fitted to the linear pooled priors, we detected similar results as for the analysis using linear pooled priors. Comparable to the analyses with both linear and logarithmic pooled priors, maternal and adolescent internalizing symptoms at T1 predicted maternal positive interaction behavior at T2 (*M*_*maternal*_ = −0.23, 95% HPD = [−0.38,−0.07]; *M*_*adolescent*_ = −0.20, 95% HPD = [−0.38,−0.03]). However, we found no evidence for associations between maternal or adolescent interaction behavior and their subsequent internalizing symptoms, which is in line with the linear pooled priors, but only partially in line with the logarithmic pooled priors. As in the other analyses using linear and logarithmic pooled priors, maternal and adolescent internalizing symptoms at T1 predicted their own respective symptoms at T2. No indirect effects were found. Further sensitivity analyses with default priors, which relied on prespecified non-informative priors, yielded the same conclusions as the analysis with fitted normal priors.

**FIGURE 4:**
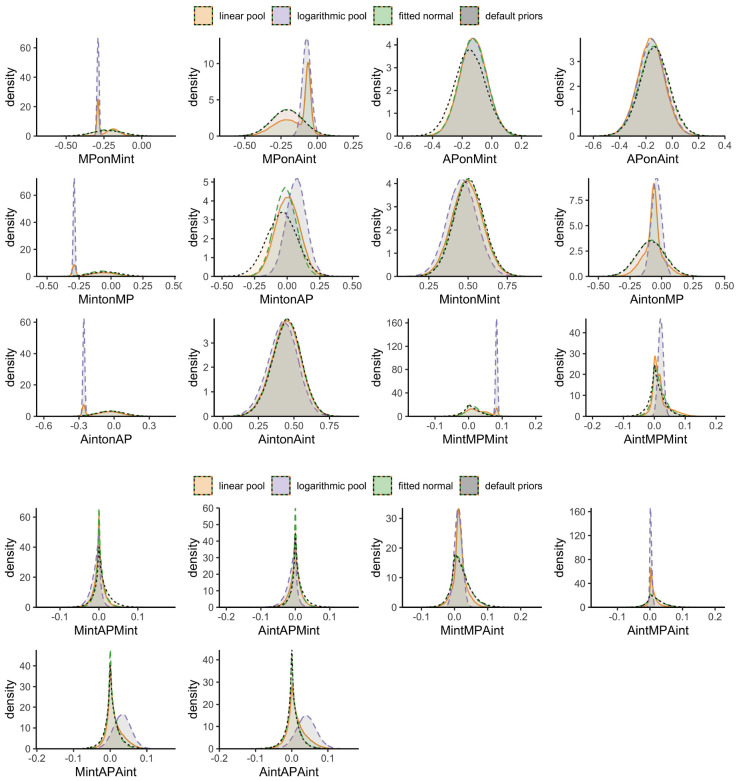
Posterior distributions of the final results involving positive interaction behavior; linear pooled priors are displayed in orange, logarithmic pooled priors in light-purple, fitted normal priors in green, and default priors in gray.

Examining the posterior samples per parameter (see Figure 4) indicated that the linear pooled priors affected posterior samples for some parameters in such a manner that they became bimodal. For example, the posterior distribution of the association between maternal internalizing behavior and subsequent maternal positive interaction behavior (MPonMint) shows that there was some strong evidence from previous studies. This previous evidence supports an effect that is larger than what is found in the current data, as indicated by the shift in modes as compared to the analyses with default priors. On the other hand, for the association between adolescent internalizing symptoms and subsequent maternal positive interaction behavior (MPonAint), the posterior distribution still reflects some strong evidence from previous studies for an effect smaller than found in the data.

#### Negative Interaction Behavior

The results for the negative interaction behavior model as analyzed with the three different prior settings are provided in Table 6. Based on the analysis with linear pooled priors, we found that higher levels of maternal, but not adolescent internalizing symptoms predicted higher levels of subsequent maternal, but not adolescent negative interaction behavior 1 year later (*M* = 0.23, 95% HPD = [0.06,0.39]). In turn, maternal negative interaction behavior at T2 predicted adolescent, but not maternal internalizing symptoms 1 year later at T3 (*M* = 0.11, 95% HPD = [0.01,0.23]). There was limited evidence that adolescent negative interaction behavior at T2 predicted their own or their mothers’ internalizing symptoms at T3, 1 year later. Although for these associations the 95% HPD included both positive and negative values, the values were mostly positive. This indicates that there was more probability toward a positive effect, but still some probability that the effect was negative. Maternal negative interaction behavior mediated the association between maternal and adolescent internalizing symptoms, as indicated by the 95% HPD of the indirect effect that was completely positive (*M*_*indirect*_ = 0.03, 95% HPD = [0,0.06]). This suggests that higher levels of maternal internalizing symptoms predicted higher levels of adolescent internalizing symptoms 2 years later through increased maternal negative interaction behavior. No other indirect effects were found.

**TABLE 6:** Parameter estimates using different prior settings for Model B.

	**Linear pool priors**			**Logarithmic pool priors**			**Normal fitted to linear pool priors**			**Default priors**		
**Parameter**	**Mean**	**95% HPD**		**Mean**	**95% HPD**		**Mean**	**95% HPD**		**Mean**	**95% HPD**	
MNonMint	0.23	0.06	0.39	0.23	0.08	0.39	0.22	0.06	0.38	0.19	0.01	0.37
MNonAint	0.04	–0.10	0.19	0.04	–0.08	0.16	0.04	–0.15	0.22	0.04	–0.15	0.23
ANonMint	0.10	–0.06	0.27	0.10	–0.06	0.27	0.10	–0.06	0.27	0.11	–0.07	0.30
ANonAint	0.13	–0.04	0.30	0.13	–0.04	0.30	0.12	–0.05	0.29	0.11	–0.06	0.29
MintonMN	0.11	–0.07	0.29	0.29	0.28	0.30	0.08	–0.07	0.23	0.03	–0.14	0.19
MintonAN	0.11	–0.01	0.23	0.07	–0.05	0.18	0.11	0	0.23	0.22	0.06	0.38
MintonMint	0.49	0.34	0.64	0.46	0.30	0.61	0.49	0.34	0.65	0.49	0.34	0.65
AintonMN	0.11	0.01	0.23	0.10	0.04	0.16	0.11	–0.04	0.27	0.11	–0.05	0.27
AintonAN	0.20	–0.02	0.26	0.26	0.25	0.27	0.10	–0.06	0.26	0.10	–0.06	0.26
AintonAint	0.44	0.27	0.60	0.43	0.26	0.59	0.45	0.28	0.61	0.45	0.28	0.61
AintMNMint	0	–0.01	0.03	0.01	–0.02	0.05	0	–0.02	0.03	0	–0.02	0.02
AintANMint	0.01	–0.01	0.04	0.01	–0.01	0.03	0.01	–0.01	0.04	0.02	–0.01	0.08
MintMNAint	0.03	0	0.06	0.02	0	0.05	0.02	–0.01	0.07	0.02	–0.01	0.07
MintANAint	0.02	–0.01	0.06	0.03	–0.02	0.07	0.01	–0.01	0.04	0.01	–0.01	0.05

*M, maternal; A, adolescent; int, internalizing; P, positive interaction behavior; N, negative interaction behavior; on, describes the direction of regression; indirect effects are reported in direction of the associxation (e.g., MintMNAint describes the indirect effect from maternal to adolescent internalizing symptoms via maternal negative interaction behavior).*

The analyses with logarithmic pooled priors again demonstrated generally similar results. Higher levels of maternal, but not adolescent internalizing symptoms at T1 predicted higher levels of maternal negative interaction behavior at T2 (*M* = 0.23, 95% HPD = [0.08,0.39]). Maternal negative interaction behavior at T2 in turn predicted adolescent internalizing symptoms (*M* = 0.10, 95% HPD = [0.04,0.16]) and, in contrast to the linear pooled priors, also maternal internalizing symptoms at T3 (*M* = 0.29, 95% HPD = [0.28,0.30]). Higher levels of adolescent negative interaction behavior at T2 further predicted higher levels of subsequent adolescent internalizing symptoms at T3 as indicated by the completely positive 95% HPD (*M* = 0.26, 95% HPD = [0.25,0.27]), which contrasts with the analysis using linear pooled priors. For all other direct associations, the 95% HPD included both positive and negative values. Similar to the linear pooled priors, we detected evidence for an indirect effect from maternal to subsequent adolescent internalizing symptoms through increased maternal negative interaction behavior (*M*_*indirect*_ = 0.02, 95% HPD = [0.00,0.05]).

Based on the analysis with fitted normal priors, we found slightly different results. While maternal internalizing symptoms at T1 also predicted maternal negative interaction behavior 1 year later at T2 (*M* = 0.22, 95% HPD = [0.06,0.38]), there was only limited evidence that maternal negative interaction behavior predicted subsequent adolescent internalizing symptoms at T3 as the posterior distribution was wider and the 95% HPD thus included positive and negative values (*M* = 0.11, 95% HPD = [−0.04,0.27]. However, adolescent negative interaction behavior predicted maternal internalizing symptoms 1 year later at T3 (*M* = 0.11, 95% HPD = [0.00,0.23]). No indirect effects were found in this analysis. Further sensitivity analyses using default priors again yielded the same conclusions as the normal priors fitted to the linear pool. For the association between adolescent negative interaction behavior at T2 and subsequent maternal internalizing symptoms at T3, the effect size doubled in size compared to the linear pool, logarithmic, and fitted normal priors. The 95% HPD was even further from 0 (*M* = 0.22, 95% HPD = [0.06,0.38]), indicating stronger evidence that negative behaviors of adolescents predicted internalizing symptoms in mothers.

**FIGURE 5:**
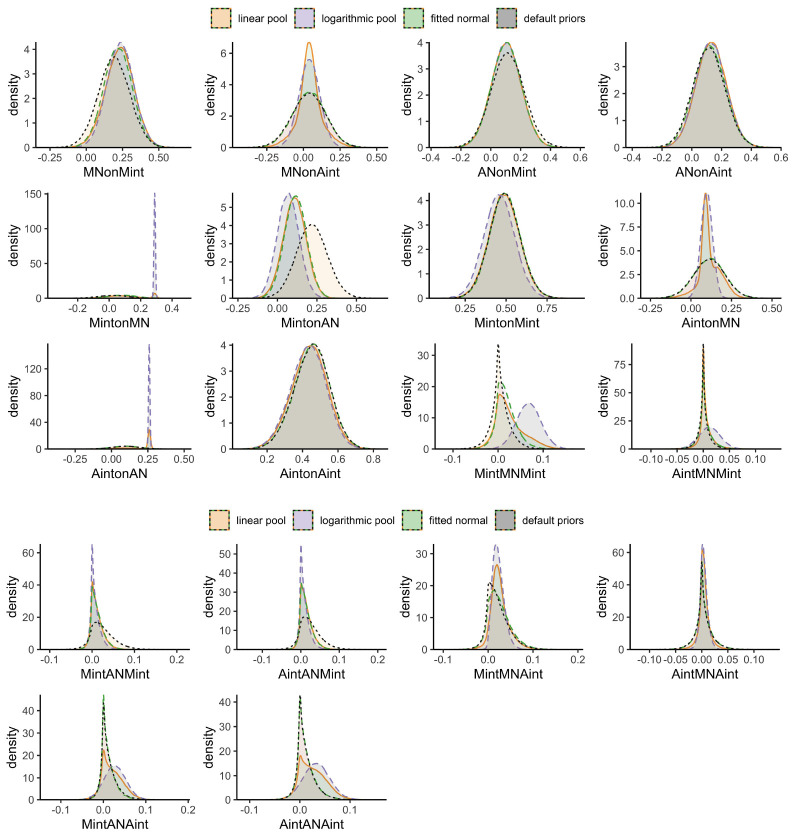
Posterior distributions of the final results involving negative interaction behavior; linear pooled priors are displayed in orange, logarithmic pooled priors in light-purple, fitted normal priors in green, and default priors in gray.

Some deviations between the results above stand out. For example, even though the mode of the pooled priors is closer to zero than the data (see Figure 5; AintonMN) maternal negative interaction behavior at T2 predicted adolescent internalizing symptoms at T3 with both pooled priors, but not with the fitted normal and default priors. Apparently, the density in the region slightly above 0 was so high that 0 was excluded from the 95% HPD for the pooled priors. On the other hand, adolescent negative interaction behavior at T2 only predicted maternal internalizing symptoms at T3 with default priors, suggesting that the prior for this parameter had a higher probability in the region around zero than our data. The posterior distribution of the association between adolescent negative interaction behavior and subsequent adolescent internalizing symptoms seemed strongly affected by the prior distribution as well, as there was a small region with extremely high probability (i.e., a spike) in the posterior around 0.25 in the analyses using linear and logarithmic pooled priors (see Figure 5; AintonAN); the 95% HPD of the linear pooled results, however, still included 0.

## Discussion

The present study used Bayesian estimation with systematically obtained results from previous studies and systematically defined prior weights, following three prior aggregation methods. The illustrative empirical research question behind this analysis concerned the mediation of bidirectional associations between maternal and adolescent internalizing symptoms from early to mid-adolescence by mother-adolescent positive and negative interaction behavior. We retrieved 47 effect sizes from 9 studies that provided information on some of the relevant parameters of our model and were thus integrated into our analyses.

### Empirical Discussion: The Mediating Role of Mother-Adolescent Interaction Behavior

Consistent with theoretical and empirical evidence that internalizing symptoms can lower maternal positive interaction behavior toward their children (Simons et al., 1993; Goodman and Gotlib, 1999; Lovejoy et al., 2000; McCabe, 2014), the distributions consistently showed that higher levels of maternal internalizing symptoms predicted lower levels of their own, but generally not adolescent positive and negative interaction behavior in the following year. Mothers with increased internalizing symptoms might be emotionally unavailable, easily irritated, and unable to sensitively respond to their children’s needs, which can suppress encouraging or nurturing behaviors and exacerbate hostile, rejecting behaviors in subsequent interactions with their children (Lovejoy et al., 2000). As maternal internalizing symptoms can disrupt interactional processes between mothers and adolescents, they are likely to drive relationship erosion in the long term (Coyne et al., 1991; Meeus, 2016). Interestingly, although the analyses using linear and logarithmic pooled priors suggested a clear negative association from adolescent internalizing symptoms to maternal positive interaction behavior as well, our findings generally provided only little evidence for the theoretical propositions that adolescent internalizing symptoms disrupt interactions in the family (Sheeber et al., 2001; Berg-Nielsen et al., 2002).

Despite theoretical propositions and empirical findings that less positive and more negative mother-adolescent interaction behavior predict adolescent internalizing symptoms (McLeod et al., 2007a,b; Yap et al., 2014; Pinquart, 2017), we found that maternal or adolescent internalizing symptoms predicted later mother-adolescent interaction behavior more often than that mother-adolescent interaction behavior predicted later internalizing symptoms. This is in line with one of the few mediation studies that found associations between maternal internalizing symptoms and observed maternal interaction behavior, but not between interaction behavior and adolescent internalizing symptoms (van Doorn et al., 2016). One possible reason for this finding may be that mother-adolescent interaction behavior is more likely to influence immediate, short-term emotions in mothers or adolescents. While particularly maternal internalizing symptoms may have long-lasting effects, maladaptive interactions may exert their effects at a shorter time interval than we could detect with annual assessments. Alternatively, highly negative and less positive interactions between mothers and adolescents are quite common in early to mid-adolescence as mother-adolescent conflicts become more intense (Hadiwijaya et al., 2017). It is possible that because such behaviors are relatively typical during this time in adolescence, they are experienced as tied to that specific interaction and thus do not directly influence adolescent mood in the long term.

The limited evidence that we found for the associations between mother-adolescent interaction behavior and later internalizing symptoms concerned mainly negative interaction behavior in the analyses using linear and logarithmic pooled priors. This may be expected given that the impact of negative events and emotions is generally stronger than the impact of positive events or emotions (Baumeister et al., 2001). Although the effect sizes were generally comparable in all analyses, using different informative priors yielded somewhat different conclusions based on the distributions and credibility intervals. Together with the detected indirect effect that maternal negative interaction behavior mediated the associations between maternal internalizing and subsequent adolescent symptoms using linear pooled and logarithmic priors, however, they suggest that negative interaction behavior may play a role in the transmission of internalizing symptoms. Hostile behaviors might make interaction partners feel rejected and helpless, undermine their self-esteem, and elicit negative self-evaluations, which might in turn increase their risk for internalizing symptoms in the long-term (Gottman et al., 1997; Garber and Flynn, 2001). Interestingly, we also found that decreased maternal positive interaction behavior mediated the associations between adolescent internalizing symptoms and subsequent maternal internalizing symptoms, but this indirect effect was only evident using logarithmic pooled priors.

The different conclusions using different priors also warrant caution. Specifically, they suggest that our data contrasts with previous findings. In the linear pooled prior distribution, we indeed detected two spikes toward a positive distribution for the associations from maternal negative interaction behavior to later adolescent internalizing symptoms, whereas the logarithmic pooled priors suggested one extreme dense, narrow distribution closer to zero and the fitted normal priors indicated a similar, but flat distribution. The detected spikes in the linear and logarithmic pooled priors resulted from information found in previous studies, which drove these conclusions, whereas associations that we only detected with fitted normal and default priors suggested that our data provided stronger evidence than previous findings. Using different approaches to define informative priors allowed us to compare their impact on the results and evaluate the robustness of our conclusions. Once we updated the information collected in previous studies with our new data, the posterior distributions shifted to a varying degree depending on how we specified the priors. Differences between the posteriors were particularly pronounced when our data strongly diverged from previous studies. While the posteriors generated from logarithmic pooled priors were strongly influenced by previous data and thus only shifted little compared to the prior distributions, the linear pooled priors often resulted in bimodal distributions that reflected the discrepancy between previous and new data. These differences in priors and previous compared to new data emphasize that for some associations, we may not yet have enough evidence to draw final conclusions.

### The Role of Different Informative Priors

While we were able to include a range of findings relating to our model parameters, these studies reflected our own study’s design to a varying degree and might thus introduce potential bias (Hobbs et al., 2011; Viele et al., 2014). Each included study provides a varying amount of relevant information and certainty, which is essential to take into account when specifying informative priors. How much a previous study contributes, depends on the focus and methodological considerations of the specific study. A weighting scheme therefore needs to be tailored to each new study’s purpose and design. To avoid bias, such as increased subjectivity, it is important to engage content experts who can judge the relevance of weighting aspects and justify all decisions transparently in an accessible logbook (Zondervan-Zwijnenburg et al., 2017). Therefore, we involved content experts to design a weighting scheme and scoring system that allowed us to consider each study’s specific contribution with respect to our data. Our illustrative example represented a longitudinal, multi-method design, which constituted the core of our weighting scheme. As cross-sectional studies cannot be used to disentangle the temporal order of associations, they provided only weak evidence for our parameters. Similarly, longitudinal studies that did not control for previous levels of psychopathological symptoms at an earlier point in time are not useful to measure change, and therefore received less weight as well. While a weighting scheme is an essential tool to combine findings from more or less comparable studies, it needs to be carefully constructed and reviewed to avoid inaccurate inferences and conclusions. In this study, we followed recommendations, such as including experts for the composition of the weighting scheme or the estimation of the weights (e.g., Zondervan-Zwijnenburg et al., 2017), which can further help to reduce subjectivity. Instead of weights based on the match between previous studies and the design of the study at hand, weights can also be based on optimality criteria (e.g., maximum entropy, minimum Kullback-Leibler divergence) or modeled by means of a prior on the weights (e.g., de Carvalho et al., 2020). These methods do not take the content of studies into account, which can be regarded their strength because of increased objectivity, but also their weakness because previous studies are not valued based on criteria that are considered important by experts.

Our statistical evaluation showed that analyses based on linear pooled priors may suffer from estimation problems (i.e., insufficient convergence and precision), where other prior specifications do not show the same issues. Furthermore, the prior predictive distributions were comparable across prior specification methods, except for the default prior, which does not produce a meaningful predictive distribution. Generally, we found that the posterior distributions based on the analyses with linear pooled priors displayed bimodal distributions and strong spikes in multiple occasions. The posteriors resulting from the logarithmic pooled priors were spiked and highly driven by the previous information as confirmed by the low associated shrinkage. In the current study, two studies (i.e., Pinquart, 2017; Milan and Carlone, 2018) caused all spikes. These studies reported estimates with extremely small (standardized) standard errors, thus strengthening the evidence for these effects. While Pinquart (2017) conducted a longitudinal meta-analysis on the associations between parental behaviors and adolescent internalizing symptoms with over 1,000 included studies, Milan and Carlone (2018) investigated actor and partner effects in how mother and adolescent internalizing symptoms predicted maternal and adolescent behaviors during an interaction task. Both studies provide important information for our analyses, but do not precisely reflect our study design. Specifically, Pinquart’s meta-analysis also included (young) children and reported parental behaviors. Milan and Carlone, on the other hand, only sampled adolescent girls, who have been found to show higher levels of internalizing symptoms (Zahn-Waxler et al., 2008) and to be more sensitive to interpersonal experiences than adolescent boys (Flook, 2011). The design differences were taken into account by using power priors based on a systematic weighting scheme. In the current study, we did not lower study weights based on the specificity of the results. From a perspective of building cumulative knowledge, that would be a questionable practice. From a more pragmatic perspective, however, it may be sensible to downweigh information that appears unreasonably specific. For example, when expert elicitation is used to form prior distributions, it is suggested that the analyst decides to exclude an expert’s distribution if their probability density is too narrow (de Carvalho et al., 2020).

Linear pooled priors integrate all available literature to its full avail and consider the influence of potentially differing previous findings. These distributions allow – or even demand – researchers to examine extreme or varying findings and discuss their data more specifically in relation to the literature. In this manner, the linear pooled prior and its associated posterior may also provide directions for future research. However, a multimodal posterior distribution may also render it difficult to interpret the findings directly. Furthermore, extra caution is warranted to establish sufficient convergence and precision.

Logarithmic pooled priors reflect an updating process of previous studies. As such, they are closely tied to the idea of building cumulative knowledge. In the current study, the specificity of some of the previous results overruled other previous findings and the current data in the posterior. However, this does not disqualify the logarithmic pooling procedure in general, nor in this case specifically. The posterior still represents our updated previous knowledge.

An alternative to downweighing previous results based on their extreme specificity, is to fit normal distributions to the linear pooled previous information. Similar to logarithmic pooled priors, fitted normal distributions behave well during Bayesian estimation and, similar to linear pooled priors, use previous information to inform the analyses. Particularly if previous research is scarce, contradictory or only few studies are sampled, fitted normal distributions are useful to specify informative priors without overemphasizing the effect of one individual study. Fitted normal priors are best suited when it can be assumed that the previous results are random samples from an underlying normal distribution, or when the analyst considers it a pragmatic midway between the more informative pooled and default priors.

In contrast to informative priors, default priors neglect previous knowledge about how mother-adolescent interaction behavior mediate the associations between maternal and adolescent internalizing symptoms. The predictive distribution clearly showed that default priors are highly unspecific with regards to expected future data. The associated shrinkage confirmed that the observed data completely overruled the unspecific previous information. For default priors, this behavior is desired. Previous studies, however, have shown that the use of default, non-informative priors may strongly bias the results and decrease estimation accuracy, particularly in small samples (Smid et al., 2019; Zitzmann et al., 2020).

### Strengths, Limitations, and Implications

This study applied Bayesian estimation with informative priors to examine in an illustrative example whether observed mother-adolescent interaction behavior underlies the longitudinal associations between maternal and adolescent internalizing symptoms from early to mid-adolescence. Using a novel, comprehensive approach in which we first systematically quantified previous study findings in a meta-analytic design and then used this previous knowledge as input for the analyses allowed us to draw more precise conclusions about the potential mediating role of mother-adolescent interaction behavior. Such a strategy exceeds a pure meta-analytical approach, because it allowed us to incorporate existing information from a wide variety of studies that resemble our present study to varying degrees. Meta-analyses provide good starting points for new Bayesian analyses. Previous studies generate and raise new research questions, and Bayesian estimation with informative priors allows for a cumulative approach that does not ignore existing knowledge, but gradually updates it. This way, existing knowledge will be integrated into the empirical process. Particularly when previous research is scarce or when new studies are needed to address important limitations of previous research, including prior distributions can help to further cumulate knowledge. In our study, information was available on only some parameters, but not on the complete mediation model that we aimed to test. While the limited previous information was not sufficient to perform a meaningful meta-analysis, we were able to use the existing information to conduct new analyses that addressed previous limitations or remaining questions and integrated previous knowledge. By using three different priors, we were further able to show the robustness of our results across different approaches.

Despite these strengths, this study had some limitations with respect to the empirical mediation analysis. First, we only observed mother-adolescent interaction behavior at one time during early to mid-adolescence. While this approach allowed us to reduce the complexity of our model to fit our sample size, summary scores may not accurately reflect the processes that occur during the interactions between mothers and adolescents. It may be important to not only examine which average behaviors mothers and adolescents show during interactions, but also how these behaviors mutually influence each other on a moment-to-moment basis.

Second, a full longitudinal mediation approach would further require the assessment of all variables at each time point to account for the stability of not only internalizing symptoms across time, but also the stability of interaction behavior as well as concurrent associations between interaction behavior and internalizing symptoms. Due to the limited sample size in our data (*N* = 102 mother-adolescent dyads), we had insufficient information to inform a three-wave fully recursive model, which would have been ideal.

Third, longitudinal studies rarely employ the same time intervals between measurements, which renders comparing the findings from these studies difficult. Parameter estimates often depend on the time interval that was used (e.g., Gollob and Reichardt, 1987) as the underlying processes that measure change on a micro-scale, such as moment-to-moment or day-to-day, can differ from those on a macro-scale, such as year-to-year (Ebner-Priemer and Trull, 2009; Voelkle et al., 2012; Hollenstein et al., 2013). Consequently, studies with varying time scales might result in different conclusions that are not directly comparable. In our study, we tried to address time dependency by adding additional weight to studies that incorporated the same time interval as we did. However, we were only able to include few longitudinal studies, of which none received this additional weight. Future studies that aim to incorporate more, or exclusively, longitudinal studies might consider continuous rating options, such as continuous-time modeling or continuous-time meta-analytical procedures that allow to account for the effect of time more precisely (e.g., Van Montfort et al., 2018; Kuiper and Ryan, 2020). Another option could be to include a selection of varying weighting schemes and subsequently evaluate how different rating decisions affect the results. However, these approaches were beyond the scope of our empirical illustration.

In this study, we made use of differently composed informative priors to compare their effects on the posterior distributions. While our approach allowed us to systematically specify and use informative priors for the analyses of our data, quantifying, and weighing each previous study in such a systematic way requires a substantial amount of time and effort. If taken seriously, the task is equivalent to conducting a weighted meta-analysis with the additional benefit of including information from studies that resemble the present study to a varying extend. By allowing researchers to integrate new data and evaluate novel research questions using existing knowledge, this approach moves beyond where meta-analytical methods usually end and allows for knowledge to further cumulate over time.

As such, Bayesian estimation with informative priors can address important shortcomings of current empirical practices and serve the goal of empirical research to generate scientific growth of knowledge. Nevertheless, in such a systematic approach it is essential to effectively use previous information for Bayesian estimation. Knowing the literature and making informed decisions about relevant studies allows researchers to consider the most suitable approach to defining priors for their specific situation. This is important to avoid incorporating information from only one individual sample, while years of research already established well-grounded expectations. Focusing on the 95% HPD for hypothesis testing, our results did not detect many differences between the use of pooled or fitted normal priors.

How results from multiple previous studies on the same parameter should be included in the associated prior depends on theoretical considerations: Should the prior reflect the previous results as they are (linear pool), be an update of previous results (logarithmic pool), or be considered a set of random samples from an underlying normal distribution (normal fitted to the linear pool)? The differences between the approaches are emphasized when results diverge across previous studies: Are all results plausible and can they coexist in the prior distribution (linear pool), is only the consensual part plausible (logarithmic prior), or is there an underlying truth that is best resembled by a fitted normal distribution (fitted normal)? Additionally, pragmatic considerations can be taken into account. For example, the logarithmic pool is a theoretically sound (Bayesian) approach to aggregate multiple previous results that will emphasize consensual values, but extremely specific results from previous studies lead it to exclude large portions of the sample space. In the same situation, the posterior distributions based on the linear pooled priors do not exclude the observed values. However, the bimodal results that can result from diverging previous findings are difficult to interpret substantively. In these cases, a prior distribution like the fitted normal may be preferred, as it eliminates most of the impact of studies with high density when more studies contribute to the previous information.

## Conclusion

Testing a comprehensive model that includes mediation effects requires a large sample size to detect small-to-moderate effects that are common in social science. Typically, studies including longitudinal, observational designs include only relatively small samples as they are time-consuming, costly, demand more of the participants, and face recruitment difficulties, such as dropout. Attempting to estimate complex models with traditional analytical techniques can result in estimation problems as well as inaccurate parameter estimates (e.g., van de Schoot et al., 2017), and thus limit the conclusions that can be drawn from such models. Furthermore, by using informative priors, we gain insight into how our data relate to the results from previous studies.

The findings of our study indicated that posterior distributions were generally stable across different prior distributions with differing levels of existing knowledge on the associations between mother-adolescent interaction behavior and internalizing symptoms. Specifically, we consistently found that even though mother-adolescent interaction behavior might play a relatively limited role in the transmission of internalizing symptoms from early to mid-adolescence, particularly negative interaction behavior might still be relevant. Nevertheless, the choice of prior aggregation did alter the results for some parameters and may well make a difference in other studies. Researchers should carefully consider how to aggregate previous results into one prior distribution, and always conduct sensitivity analyses to demonstrate if the results hold with different prior specifications. As illustrated by our example, using Bayesian estimation with informative priors offers a great opportunity to use accumulated knowledge to increase the precision of our outcomes. If conducted thoroughly, the approach equals and moves beyond where a weighted meta-analysis usually ends as it not only quantifies previous knowledge, but also integrates new data into a cumulative process. Such precision and accumulation of knowledge is important in moving empirical science forward, but also in informing therapeutic programs that aim to prevent or reduce adolescent internalizing symptoms by targeting often proposed risk factors, such as maladaptive interaction behavior between mothers and adolescents.

## Data Availability Statement

The datasets presented in this study can be found in online repositories. The names of the repository/repositories and accession number(s) can be found below: Data Archiving and Networked Services (DANS): https://easy.dans.knaw.nl/ui/datasets/id/easy-dataset:113721; doi: 10.17026/dans-zrb-v5wp.

## Ethics Statement

The studies involving human participants were reviewed and approved by Medical Ethical Committee of the University Medical Center Utrecht (protocol number: 05/159-K). Written informed consent to participate in this study was provided by all adolescents and their parents.

## Author Contributions

SS conceived of the study and design, which was further refined by SN, AO, SB, and WM. SS drafted the manuscript, MZ-Z drafted the statistical sections. MZ-Z and DV verified the analytical methods and performed the statistical analyses. All authors discussed the results, critically revised the manuscript, and approved its final version.

## Conflict of Interest

The authors declare that the research was conducted in the absence of any commercial or financial relationships that could be construed as a potential conflict of interest.

## Footnotes

1 Note that just as in multiplication in general, the order of updating is irrelevant for the final outcome.

2 As starting year, we chose the date of the last updated search of the meta-analyses.

3 If only the standard error of the unstandardized effect was present, we multiplied that standard error with the standard deviation of the independent variable and divided by the standard deviation of the dependent variable. If a t-statistic was present, the standard error was computed by dividing the standardized effect by t. If a confidence interval for the standardized effect was provided, the difference between upper and lower limit was divided by 2 and by 1.96.

4 brms includes a function that applies multiple imputation and the aggregation of results in one step, but we did not use it for reasons of comparability between methods.
