# Supplementary material for: Systematically Defined Informative Priors in Bayesian Estimation: An Empirical Application on the Transmission of Internalizing Symptoms Through Mother-Adolescent Interaction Behavior
Source: Front Psychol. 2021 Mar 24;12:620802. doi: 10.3389/fpsyg.2021.620802 (PMC8024698; doi:10.3389/fpsyg.2021.620802)
Supplement: Supplementary file 2 [file Table_1.DOCX]

# **Review of Prior Knowledge on the Role of Mother-Adolescent Interaction Behavior**

Several studies have been conducted to support the theoretically proposed associations of maternal and adolescent internalizing symptoms with mother-adolescent interaction behavior. These previous findings provide the core input for informative priors that can be used to inform novel analyses and thus increase the precision of posterior distributions. In constructing such strongly defined informative priors, methodological study differences need to be taken into account and weighed according to how well they represent and match the present study’s design and how strongly they support the theoretically proposed hypotheses. Effectively using the quantified information to increase the precision of the results thus requires informative priors that are specified systematically and transparently (van de Schoot et al., 2015; Zondervan-Zwijnenburg et al., 2017). In the following, we systematically review and discuss the support from previous meta-analytic and longitudinal studies as input for our informative priors.

**Support from meta-analytic studies.** In line with theoretical models, meta-analytical studies indeed found that mothers with more internalizing symptoms used less positive and more negative interaction behaviors towards their children than mothers with fewer internalizing symptoms (Lovejoy et al., 2000; McCabe, 2014). In turn, children whose mothers used less positive interaction behavior and more negative interaction behavior showed higher levels of internalizing symptoms than children whose mothers used less positive and more negative interaction behavior (McLeod et al., 2007a; McLeod et al., 2007b; Pinquart, 2017; Yap et al., 2014). Recent longitudinal meta-analyses further found that maternal positive and negative interaction behavior predicted subsequent child internalizing symptoms (Pinquart, 2017; Yap et al., 2014), while prior child internalizing symptoms also predicted lower levels of subsequent mother-child positive, but not negative interaction behaviors (Pinquart, 2017).

As meta-analyses quantify a large body of empirical research, they provide reliable information for the construction of informative priors. However, drawing conclusions from these meta-analyses to our specific developmental period and design is also difficult. First, most meta-analyses included a broad age range from infancy throughout adolescence. Mother-child interactions change in structure and function over time. In adolescence, interactions between mothers and their children increase in conflict as adolescents negotiate their growing independence (Laursen and Collins, 2009) and experience more intense, fluctuating emotions than they did in childhood (Larson et al 1996; Larson et al 2002). Mother-child interactions during early childhood can thus not adequately reflect these changing interactions during adolescence. Second, most meta-analyses included both self-reports and observations to assess mother-adolescent interaction behavior. However, self-reports and observations of interaction behaviors are usually only weakly associated (Herbers et al., 2017; Moens et al., 2018). Although observational assessments of parent-adolescent interactions are more objective and valid to reflect interactions between mothers and adolescents, and are thus clearly preferred in the field (Gardner, 2000; Herbers et al., 2017; Lovejoy et al., 2000; Repetti et al., 2015), to date longitudinal multi-method studies are still scarce. Third, all meta-analyses on the associations between maternal internalizing symptoms and mother-adolescent interaction behavior reflect concurrent associations that do not allow conclusions about temporal associations and direction of effects. As causal or temporal associations between mother-adolescent interaction behaviors and maternal as well as adolescent internalizing symptoms take time to unfold, longitudinal studies can more accurately reflect these mediation processes than cross-sectional studies (Maxwell & Cole, 2007; Kraemer et al., 2000; Gollob & Reichardt, 1987).

**Support from longitudinal studies.** Observational, longitudinal assessments in adolescence best reflect our study’s design and thus provide the strongest evidence for specifying informative priors. The few findings on longitudinal associations between self-reported maternal internalizing symptoms and observed maternal interaction behavior, or between observed maternal interaction behavior and adolescent internalizing symptoms, remain inconsistent. Maternal internalizing symptoms have been shown to predict subsequent maternal positive (Feng et al., 2007; Simons et al., 1993), but not negative interaction behavior (Feng et al., 2007). Furthermore, some studies found that maternal positive and negative interaction behavior predicted adolescent internalizing symptoms (Hofer et al., 2013; Milan et al., 2018), while other studies failed to find such associations (Feinberg et al., 2007; Schwartz et al., 2012). The only study that also tested reversed associations from adolescents to mothers found that observed adolescent positive and negative interaction behaviors did not predict subsequent maternal internalizing symptoms (Milan & Carlone, 2018). Differences in the assessments of internalizing symptoms (e.g., diagnosis vs. symptoms; depressive vs. internalizing symptoms), age ranges, and time intervals (ranging from two weeks to three years) may explain these inconsistent findings.

While the reviewed studies provide some indications that maternal positive and negative interaction behavior mediate the transmission of internalizing symptoms, only few studies tested these associations in a mediation model. These studies found that self-reported maternal negative, but not positive interaction behavior mediate the prospective association between maternal and adolescent internalizing symptoms (Elgar et al., 2007; Johnson et al., 2001; Sellers et al., 2014). Another study that also controlled for prior adolescent internalizing symptoms, however, did not find that maternal negative interaction behavior mediated the association between maternal depression history and subsequent adolescent internalizing symptoms (Frye & Garber, 2005). Although these studies support a potential mediation effect of interaction behaviors from maternal to adolescent internalizing symptoms, they did not observe interaction behaviors and thus, do not provide suitable information to construct informative priors in our study. The two studies that tested mediating effects of observed maternal interaction behavior found that maternal depressive symptoms were associated with observed maternal interaction behavior, but only one study found that both maternal positive and negative interaction behavior predicted adolescent depressive symptoms (Olino et al., 2016), whereas the other found that positive interaction behavior did not predict adolescent internalizing symptoms (Van Doorn et al., 2016). Both studies failed to detect significant mediation effects.

# **Defining priors – List of included effect sizes**

**Model A) mother-adolescent positive interaction behavior**


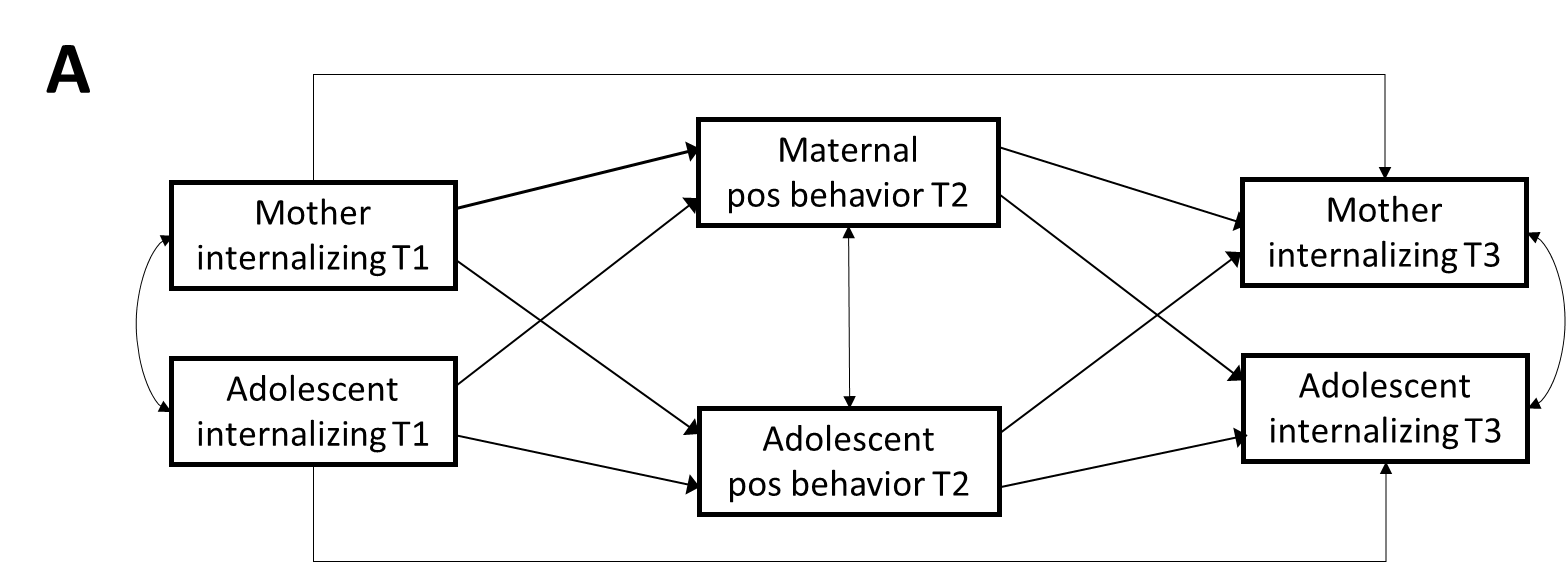


= effect sizes excluded due to insufficient information

| **Study** | **Score** | **Analysis** | **stand β** | **SE/CI** | **unstand** | **Comment** |
| --- | --- | --- | --- | --- | --- | --- |
| ***M internalizing T1 🡪 M positive interaction behavior T2*** | | | | | | |
| *Simons (1993) | 20/80 | Correlation/  Path model | *r* = −.05/  β = -.394 |  |  | No standardized SE |
| McCabe (2014) | 35/80 | Meta-analysis  MA path model | ρ = −.18/  β = −.15 | CI= −.21, −.14/  SE=.02 |  |  |
| Dietz (2008) | 25/80 | ANOVA (more vs. less dep) | B = −.21 | t(92)=−2.02 |  |  |
| Milan (2018) | 30/80 | SEM | β = −.29 | SE = .01 (unstandard); SD= 0.86 (M pos), SD= 5.56 (M int) | B = −.04 | Pos + neg mixed |
| Van Doorn (2016) | 15/80  25/80 | Correlation/  Regression | *r* = −.23  β = −.22 |  |  | No standardized SE |
| ***M internalizing T1 🡪 A positive interaction behavior T2*** | | | | | | |
| Milan (2018) | 30/80 | SEM | −.06 | SE = .01 (unstandard); SD= 0.79 (A pos); SD= 5.56 (M int) | B = −.01 | Pos + neg mixed |
| ***A internalizing T1 🡪 M positive interaction behavior T2*** | | | | | | |
| Pinquart (2017) | 40/80 | Meta-analysis | *r* = −.06 | CI=−.08; −.05 |  | controlling for interaction T1 |
| Dietz (2008) | 25/80 | ANOVA | M = 3.2  (dep, n=43)  M = 3.3  (hi risk, n=28)  M = 3.3  (lo risk, n=41) | SD = 0.5  SD = 0.5  SD = 0.5 |  | Hi risk = parental diagn of interaction behavior disorder |
| Dadds (1992) | 15/80 | MANOVA  (M= % of pos directed at child) | M = 61.9  (dep, n=18)  M = 71.6  (no-dep, n=16) | SD = 21.4  SD = 18.5 |  |  |
| Milan (2018) | 30/80 | SEM | −.16 | SE = .01 (unstandard); SD= 0.86 (M pos), SD= 5.51 (A int) | B = −.01 |  |
| ***A internalizing T1 🡪 A positive interaction behavior T2*** | | | | | | |
| Dadds (1992) | 15/80 | MANOVA | M = 28.1  (dep, n=18)  M = 27.8  (no-dep, n=16) | SD = 15.5  (dep)  SD = 20.1  (no-dep) |  |  |
| Dietz (2008) | 25/80 | ANOVA | M = 2.4  (dep, n=43)  M = 2.5  (hi risk, n=28)  M = 2.9  (lo risk, n=41) | SD = 0.6  SD = 0.4  SD = 0.4 |  | Hi risk = parental diagn of interaction behavior disorder |
| Milan (2018) | 30/80 | SEM | −.26 | SE = .01 (unstandard); SD= 0.79 (A pos), SD= 5.51 (A int) | B = −.04 |  |
| ***M positive interaction behavior T2 🡪 M internalizing T3*** | | | | | | |
| Dietz (2008) | 25/100 | ANOVA | B = −.21 | t(92)=−2.02 |  |  |
| Milan (2018) | 30/100 | SEM | −.29 | SE = .01 (unstandard); SD= 0.86 (M pos), SD= 5.56 (M int) | B = −.04 | Pos + neg mixed |
| Van Doorn (2016) | 25/100 | Regression | −.22 (sig) |  |  | No standardized SE |
| ***M positive interaction behavior T2 🡪 A internalizing T3*** | | | | | | |
| Pinquart (2017) | 60/100 | Meta-analysis | *r* = −.06 | CI=−.09; −.04 |  |  |
| Olino (2016) | 50/100 | Trajectories |  | *SE* = .79  (unstandard) | *B* =−1.28 | No standardized info |
| Schwartz (2012) | 70/100 | Path model | Dep:  −.09(girls)  −.10(boys)  Anx:  −.02(girls)  −.02(boys) | Dep (all unstand):  SE=0.77, CI= −2.54;0.49; SD=7.97 (A dep), SD=0.69 (M pos)  Anx (all unstand):  SE=0.93, CI=-2.02; 1.63; SD=8.72 (A anx) | Dep:  B= −1.03  Anx:  B= −0.20 | Dep= depression, anx=anxiety  Girls = 87 (48.9%)  Boys = 91 |
| Hofer (2013) | 55/100  80/100 | Correlation/  Path model | r= −.11 / | SE=.04 (unstandard) | B = −.08 | No standardized info |
| Milan (2018) | 60/100 | SEM | −.21 | SE = .35 (unstandard);  SD= 0.86 (M pos), SD= 5.51 (A int) | B = −1.04 | Pos + neg mixed |
| Dietz (2008) | 25/100 | ANOVA | M = 3.2  (dep, n=43)  M = 3.3  (hi risk, n=28)  M = 3.3  (lo risk, n=41) | SD = 0.5  SD = 0.5  SD = 0.5 |  | Hi risk = parental diagn of interaction behavior disorder (see comment above) |
| Dadds (1992) | 15/100 | MANOVA | M = 61.9  (dep, n=18)  M = 66.3  (no-dep, n=16) | SD = 21.4  SD = 14.9 |  |  |
| Griffith (2019) | 55/100 | Logistic regression (support + responsive-ness) | Odds ratio = 1.05 (support)  OR = 0.79  (respons) | CI=0.87, 1.26  CI=0.63, 1.00 |  |  |
| ***A positive interaction behavior T2 🡪 M internalizing T3*** | | | | | | |
| Milan (2018) | 60/100 | SEM | −.005 | SE = .53 (unstandard); SD= 0.79 (A pos); SD= 5.56 (M int) | B = −.04 | Pos + neg mixed |
| ***A positive interaction behavior T2 🡪 A internalizing T3*** | | | | | | |
| Dadds (1992) | 15/100 | MANOVA | M = 28.1  (dep, n=18)  M = 27.8  (no-dep, n=16) | SD = 15.5  (dep)  SD = 20.1  (no-dep) |  |  |
| Dietz (2008) | 25/100 | ANOVA | M = 2.4  (dep, n=43)  M = 2.5  (hi risk, n=28)  M = 2.9  (lo risk, n=41) | SD = 0.6  SD = 0.4  SD = 0.4 |  | Hi risk = parental diagn of interaction behavior disorder (see comment above) |
| Milan (2018) | 30/100 | SEM | −.26 | SE = .01 (unstandard); SD= 0.79 (A pos); SD= 5.51 (A int) | B = −.04 | Pos + neg mixed |
| ***T2 correlation: M positive interaction behavior T2 + A positive interaction behavior T2*** | | | | | | |
| Milan (2018) | 25/100 | Correlation | r = .51 |  |  | No standardized info |
| Hofer (2013) | 12.5/100 | Correlation | r=.57(T1), .65(T2) |  |  | No standardized info |

**Model B) mother-adolescent negative interaction behavior**


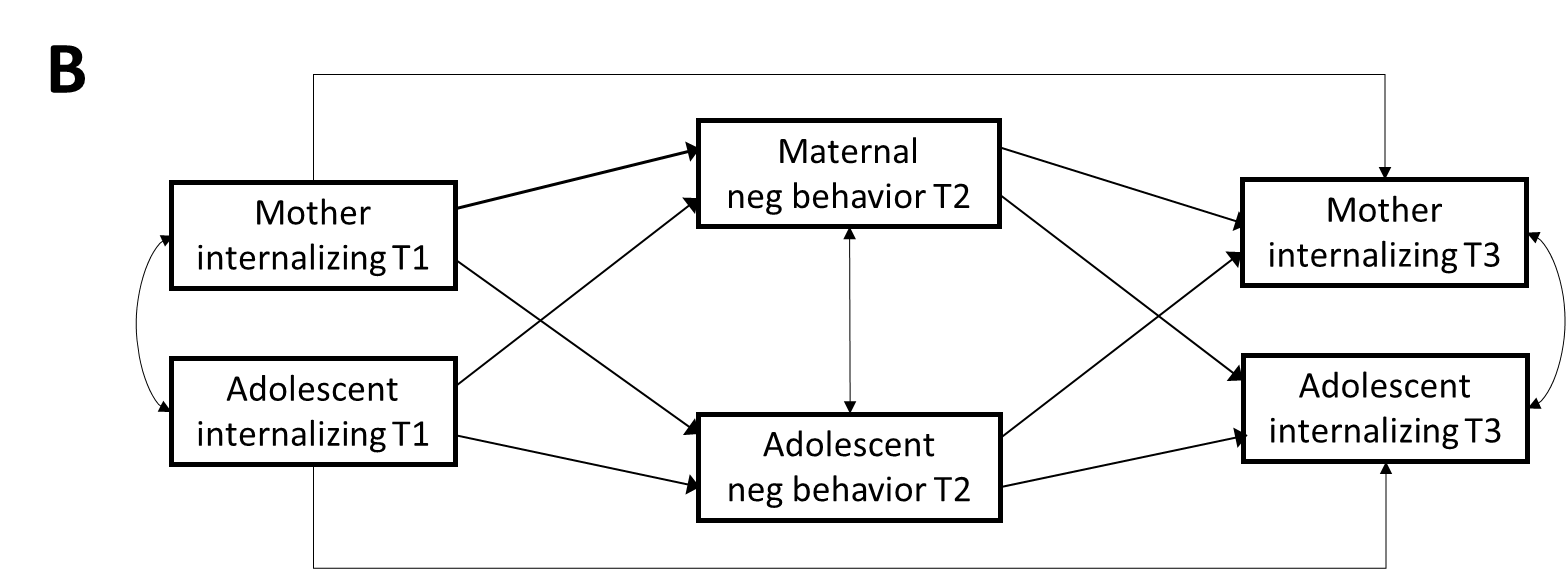


| **Study** | **Score** | **Analysis** | **stand β** | **SE/CI** | **unstand** | **Comment** |
| --- | --- | --- | --- | --- | --- | --- |
| ***M internalizing T1 🡪 M negative interaction behavior T2*** | | | | | | |
| Dietz (2008) | 25/80 | ANOVA |  | t(92)=2.36 | B = .24 | No standardized info |
| Lovejoy (2000) | 25/80 | Meta-analysis | *d* = .40 | CI= .31;.49 |  |  |
| Milan (2018) | 30/80 | SEM | β = .29 | SE = .01 (unstandard); SD= 0.54 (M neg); SD= 5.56 (M int) | B = .04 | Pos + neg mixed |
| ***M internalizing T1 🡪 A negative interaction behavior T2*** | | | | | | |
| Milan (2018) | 30/80 | SEM | .06 | SE = .01 (unstandard); SD= 0.63 (A neg); SD= 5.56 (M int) | B = .01 | Pos + neg mixed |
| ***A internalizing T1 🡪 M negative interaction behavior T2*** | | | | | | |
| Pinquart (2017) | 40/80 | Meta-analysis | *r* = .04 | CI= −.03; .11 |  | controlling for interaction T1 |
| Dietz (2008) | 25/80 | ANOVA | M = 2.2  (dep, n=43)  M = 2.1  (hi risk, n=28)  M = 2.0  (lo risk, n=41) | SD = 0.9  SD = 0.8  SD = 0.8 |  | Hi risk = parental diagn of interaction behavior disorder |
| Dadds (1992) | 15/80 | MANOVA | M = 10.3 (dep, n=18)  M = 3.9  (no-dep, n=16) | SD = 13.4  SD = 8.7 |  |  |
| Szwedo (2017) | 35/80 | Regression | .15 |  |  | No standardized SE |
| Milan (2018) | 30/80 | SEM | .16 | SE = .01 (unstandard); SD= 0.54 (M neg); SD= 5.51 (A int) | B = .01 |  |
| Asbrand (2017) | 35/80 | MANOVA | M = 3.32  (anx, n=27)  M = 2.59  (non-anx, n=27) | SD = 1.36  SD = 1.37 |  |  |
| ***A internalizing T1 🡪 A negative interaction behavior T2*** | | | | | | |
| Dadds (1992) | 15/80 | MANOVA | M = 17.8 (dep, n=18)  M = 12.1 (no-dep, n=16) | SD = 16.9  (dep)  SD = 17.8  (no-dep) |  |  |
| Jackson (2011) | 15/80 | regression |  | SE = 0.169  (unstandard) | B=−2.12 (dep = more anger) | No standardized info |
| Dietz (2008) | 25/80 | ANOVA | M = 2.7  (dep, n=43)  M = 2.3  (hi risk, n=28)  M = 2.1  (lo risk, n=41) | SD = 1.0  SD = 0.9  SD = 0.8 |  | Hi risk = parental diagn of interaction behavior disorder |
| Milan (2018) | 30/80 | SEM | .26 | SE = .01 (unstandard); SD= 0.63 (A neg); SD= 5.51 (A int) | B = .04 |  |
| Asbrand (2017) | 35/80 | MANOVA | M = 3.88  (anx, n=27)  M = 3.20  (non-anx, n=27) | SD = 1.56  SD = 1.31 |  |  |
| ***M negative interaction behavior T2 🡪 M internalizing T3*** | | | | | | |
| Dietz (2008) | 25/100 | ANOVA | B = .24 | t(92)=2.36 |  |  |
| Milan (2018) | 30/100 | SEM | .29 | SE = .01 (unstandard); SD= 0.54 (M neg); SD= 5.56 (M int) | B = .04 | Pos + neg mixed |
| ***M negative interaction behavior T2 🡪 A internalizing T3*** | | | | | | |
| Pinquart (2017) | 60/100 | Meta-analysis | *r* = .09 | CI=.07; .11 |  |  |
| Olino (2016) | 50/100 | Trajectories |  | *SE* = .81;  (unstandard) | *B* = 1.23 | No standardized info |
| Schwartz (2012) | 70/100 | Path model | Dep:  .10 (girls)  .12(boys)  Anx:  .12 (girls)  .13(boys) | Dep (all unstand):  SE=0.91, CI= −.41; 3.17  Anx (all unstand):  SE=1.10, CI= −0.37;3.93 | Dep: B=1.38  Anx: B=1.78 | Dep= depression, anx=anxiety  Girls = 87 (48.9%)  Boys = 91 |
| Hofer (2013) | 55/100  80/100 | Correlation/  Path model | Neg: r=−.08  Anger:  r=−.14 */* | SE=.06 (unstandard) | B = −.14 | No standardized info |
| Szwedo (2017) | 55/100 | Regression | .15 |  |  | No standardized SE |
| Dietz (2008) | 25/100 | ANOVA | M = 2.2  (dep, n=43)  M = 2.1  (hi risk, n=28)  M = 2.0  (lo risk, n=41) | SD = 0.9  SD = 0.8  SD = 0.8 |  | Hi risk = parental diagn of interaction behavior disorder |
| Dadds (1992) | 15/100 | MANOVA | M = 10.3 (dep, n=18)  M = 3.9  (no-dep, n=16) | SD = 13.4  SD = 8.7 |  |  |
| Milan (2018) | 60/100 | SEM | .21 | SE = .35 (unstandard); SD= 0.54 (M neg); SD= 5.51 (A int) | B = 1.04 | Pos + neg mixed |
| Weymouth (2016) | 30/100 | Meta-analysis | .26 | SE = .026 |  |  |
| Griffith (2019) | 55/100  60/100 | Logistic regression (criticism, conflict) | Odds ratio = 1.04 (criticism)  OR = 1.33  (conflict) | CI=0.87, 1.25  CI=1.10, 1.61 |  | Only for conflict also controlling for parental dep (maybe only use conflict then?!) |
| ***A negative interaction behavior T2 🡪 M internalizing T3*** | | | | | | |
| Milan (2018) | 60/100 | SEM | .005 | SE = .53 (unstandard); SD= 0.63 (A neg); SD= 5.56 (M int) | B = .04 | Pos + neg mixed |
| ***A negative interaction behavior T2 🡪 A internalizing T3*** | | | | | | |
| Allen (2006) | 50/100  70/100 | Correlation/ regression | r = .33  β = .22 |  |  | No standardized SE |
| Dadds (1992) | 15/100 | MANOVA | M = 17.8 (dep, n=18)  M = 12.1 (no-dep, n=16) | SD = 16.9  (dep)  SD = 17.8  (no-dep) |  |  |
| Jackson (2011) | 15/100 | regression |  | SE = 0.169 (unstandard) | B= −2.12 (dep= more anger) | No standardized info |
| Milan (2018) | 30/100 | SEM | .26 | SE = .01 (unstandard); SD= 0.63 (A neg); SD= 5.51 (A int) | B = .04 | Pos + neg mixed |
| ***T2 correlation: M negative interaction behavior T2 + A negative interaction behavior T2*** | | | | | | |
| Milan (2018) | 25/100 | Correlation | r = .39 |  |  | No standardized info |
| Hofer (2013) | 37.5/100 | Correlation | Neg: r=  .68(T1),  .44 (T2)  Anger: r= .47 (T1), .24(T2) |  |  | No standardized info |
| McKillop (2018) | 12.5/100 | Correlation | r = .18 |  |  | No standardized info |

**General parameters: Stability + intercepts**

| **Study** | **Score** | **Analysis** | **stand β** | **SE/CI** | **unstand** | **Comment** |
| --- | --- | --- | --- | --- | --- | --- |
| ***Stability: M internalizing T1 🡪 M internalizing T3*** | | | | | | |
| Milan (2018) | 30/100 | SEM | .79 | SE=.07 (unstandard?) | .80 | Only depression |
| Schulz et al (unpub) | 70/100 | CLPM | .27 | SE=.035;  CI=.21; .34 |  | Same data, but bigger sample (includes our sample!) |
| ***Stability: A internalizing T1 🡪 A internalizing T3*** | | | | | | |
| Allen (2006) | 60/100 | regression | β = .39 |  |  | controlling for all predictors |
| Schwartz (2012) | 20/100 | Path model | Dep:  .26 (girls) .35(boys)  Anx:  .24 (girls)  .25(boys) | Dep:  SE=.05, CI=.015; .34  Anx: SE=0.07, CI=0.11, 0.37  (all unstand) | Dep:  B=.25  Anx:  B=0.24 | Dep= depression, anx=anxiety  Girls = 87 (48.9%)  Boys = 91 |
| Milan (2018) | 30/100 | SEM | .72 | .05  (unstandard?) | B=.54 |  |
| Kim (2009) | 10/100 | Path model | .23 | t=2.59 |  | Only male adolescents |
| Schulz et al (unpub) | 70/100 | CLPM | .23 | SE=.034;  CI=.16; .30 |  | Same data, but bigger sample (includes our sample!) |
| ***T1 correlation: M internalizing T1 + A internalizing T1*** | | | | | | |
| Milan (2018) | 20/100 | SEM | r = .22 |  |  | No standardized SE |
| Connell (2002) | 60/100 | Meta-analysis | dep = .16  anx = .16 (2-18yr) | CI=.15-.17 CI=.14-.18 |  | Dep + anx = maternal dep or anx |
| Goodman (2011) | 50/100 | Meta-analysis | r = .23 | CI=.22-.24 |  |  |
| Schulz et al (unpub) | 60/100 | CLPM | .23 | SE=.050;  CI=.13; .33 |  | Same data, but bigger sample  (includes our sample!) |

# **Syntax for prior specification and analyses**

Please see the uploaded .stan files for the syntax on all analyses regarding Model A (M1; mother-adolescent positive interaction behavior) and all analyses regarding Model B (M2; mother-adolescent negative interaction behavior).
